# Development of a ternary cyclodextrin–arginine–ciprofloxacin antimicrobial complex with enhanced stability

**DOI:** 10.1038/s42003-022-04197-9

**Published:** 2022-11-12

**Authors:** Marija Vukomanovic, Lea Gazvoda, Mario Kurtjak, Jitka Hrescak, Blaž Jaklic, Laura Moya-Andérico, Maria del Mar Cendra, Eduard Torrents

**Affiliations:** 1 Advanced Materials Department, Institute Jozef Stefan, Jamova 39, Ljubljana, Slovenia; 2 International Postgraduate School of Jozef Stefan, Jamova 39, Ljubljana, Slovenia; 3 Center for Electronic Microscopy and Microanalysis (CEMM), Institute Jozef Stefan, Jamova 39, Ljubljana, Slovenia; 4 Bacterial Infections: Antimicrobial Therapies, Institute for Bioengineering of Catalonia (IBEC), The Institute of Science and Technology, Baldiri Reixac 15-21, 08028 Barcelona, Spain; 5 Microbiology Section, Department of Genetics, Microbiology and Statistics, Faculty of Biology, University of Barcelona, 643 Diagonal Ave., 08028 Barcelona, Spain

**Keywords:** Drug delivery, Antibiotics

## Abstract

Designing useful functionalities in clinically validated, old antibiotics holds promise to provide the most economical solution for the global lack of effective antibiotics, as undoubtedly a serious health threat. Here we show that using the surface chemistry of the cyclodextrin (βCD) cycle and arginine (arg) as a linker, provides more stable ternary antibiotic complex (βCD-arg-cpx). In contrast to classical less stable inclusion complexes, which only modify antibiotic solubility, here-presented ternary complex is more stable and controls drug release. The components of the complex intensify interactions with bacterial membranes and increase the drug’s availability inside bacterial cells, thereby improving its antimicrobial efficacy and safety profile. Multifunctional antibiotics, formulated as drug delivery systems per se, that take the drug to the site of action, maximize its efficacy, and provide optical detectability are envisaged as the future in fighting against infections. Their role as a tool against multiresistant strains remains as interesting challenge open for further research.

## Abstract (web summary)

Ternary cyclodextrin- arginine- ciprofloxacin complexes show improved stability and increased efficacy against *P. aeruginosa* in *Galleria mellonella* worms.

## Introduction

The dwindling of effective antimicrobials poses a very serious threat to global health in the modern world, as the World Health Organization recently pointed out^1^. Only a few novel antibiotics have reached the market in the last few decades, and no completely new class of antibiotics has been discovered since 1980^2^. With enormous costs and unpredictable and short-term benefits, the discovery of new antibiotics is not a major priority for the pharmaceutical industry^2,3^. Repurposing, reprofiling, or reusing clinically approved medicines has important advantages in time and cost over discovering new drug candidates, especially in emerging situations such as pandemics^4,5^. The critical benefits are a predictable safety profile, previous knowledge on manufacturing procedures, established testing protocols, more straightforward regulatory requirements, and shorter bench-to-market periods, among many others^6,7^. Therefore, it is not surprising that approximately one-third of all approved medicines in the last decade have been repurposed old drugs, which account for 25% of the revenue in the pharmaceutical industry^7,8^. Much of the effort in the current preclinical antibiotic pipeline is focused on modifying old antibiotics to increase their efficacy, particularly in synergy with other drugs or auxiliary nondrug components^9,10^. The main scientific challenges in achieving this are the limited penetration, efflux, and toxicity associated with high-dose treatment^9,10^.

A simple and very effective approach to redesigning old antibiotics includes the formation of instable complexes that modify properties such as solubility, stability, bioavailability, and permeability, thereby directly influencing their therapeutic outcome. In that context, cyclodextrins (CDs) are particularly applicable^11,12^. With a truncated cone structure, they have a hydrophilic shell (with 7 primary groups oriented toward the narrow edge and 14 secondary sugar hydroxyl groups oriented toward the wider edge of the cone, in the case of β-CD) and a hydrophobic core (with a carbon backbone of 7 glucopyranose units that make up the structure of β-CD) available for interactions with drug molecules^11,13,14^. Most commonly, antibiotics are formulated as inclusion complexes when the hydrophobic part of the drug interacts with the hydrophobic inner core area of the CD, which consequently increases its solubility by several times^11^. This approach has been applied to various antibiotics (β-lactams, microlides, fluoroquinolones, sulfonamides, tetracyclines, and aminoglycosides), and their minimal inhibitory concentrations (MICs) were reduced by factors from 2 to more than 100^11^. It is an enthalpy-driven process, and the host-guest CD-drug complex is in equilibrium with the free drug without chemical bonding^12^. This approach becomes even more effective if the CD excipient is combined with specially selected auxiliary components (such as hydrophilic polymers, amino acids, or hydroxyl acids) to form ternary complexes^15–18^. The hydrophobic part of the drug forms a CD-drug inclusion complex, while the hydrophilic part simultaneously undergoes an acid-base reaction with the auxiliary component to form a salt.

A good example is arginine, a basic amino acid, which forms salts with acidic drugs (e.g., naproxen, zoloprofen, oxaprozin) complexed with CD^16–18^. As a result, an important increase in the stability constant and complexation efficacy is obtained. Therefore, it is expected that combining an antibiotic with CD and arginine into a ternary complex might strongly influence its antimicrobial activity. However, only a few investigations of such complexes have been conducted thus far, among which a study on cefuroxime has shown drastically increased solubility after the formation of ternary complexes with CD and arginine^19^.

Instead of the less stable inclusion complexes usually formed to increase drug solubility, we designed a more stable antibiotic ternary β-cyclodextrin-arginine-ciprofloxacin (βCD-arg-cpx) complex, in which ciprofloxacin (cpx) is attached to the hydrophilic surface of βCD via an arginine (arg) linker. With this approach, our goal was not simply increasing the solubility of the drug as usual. Here, our synthetized complex system is importantly more stable to control antibiotic release, enable enhanced interactions with the bacterial cell wall and membranes, and provide higher permeability and availability inside the bacteria, consequently improving the efficacy of antibacterial treatment. Along with improved antimicrobial efficacy, considerable improvement in the safety profile was demonstrated.

## Results and discussion

### CD complex structure and formation

**Fig. 1:**
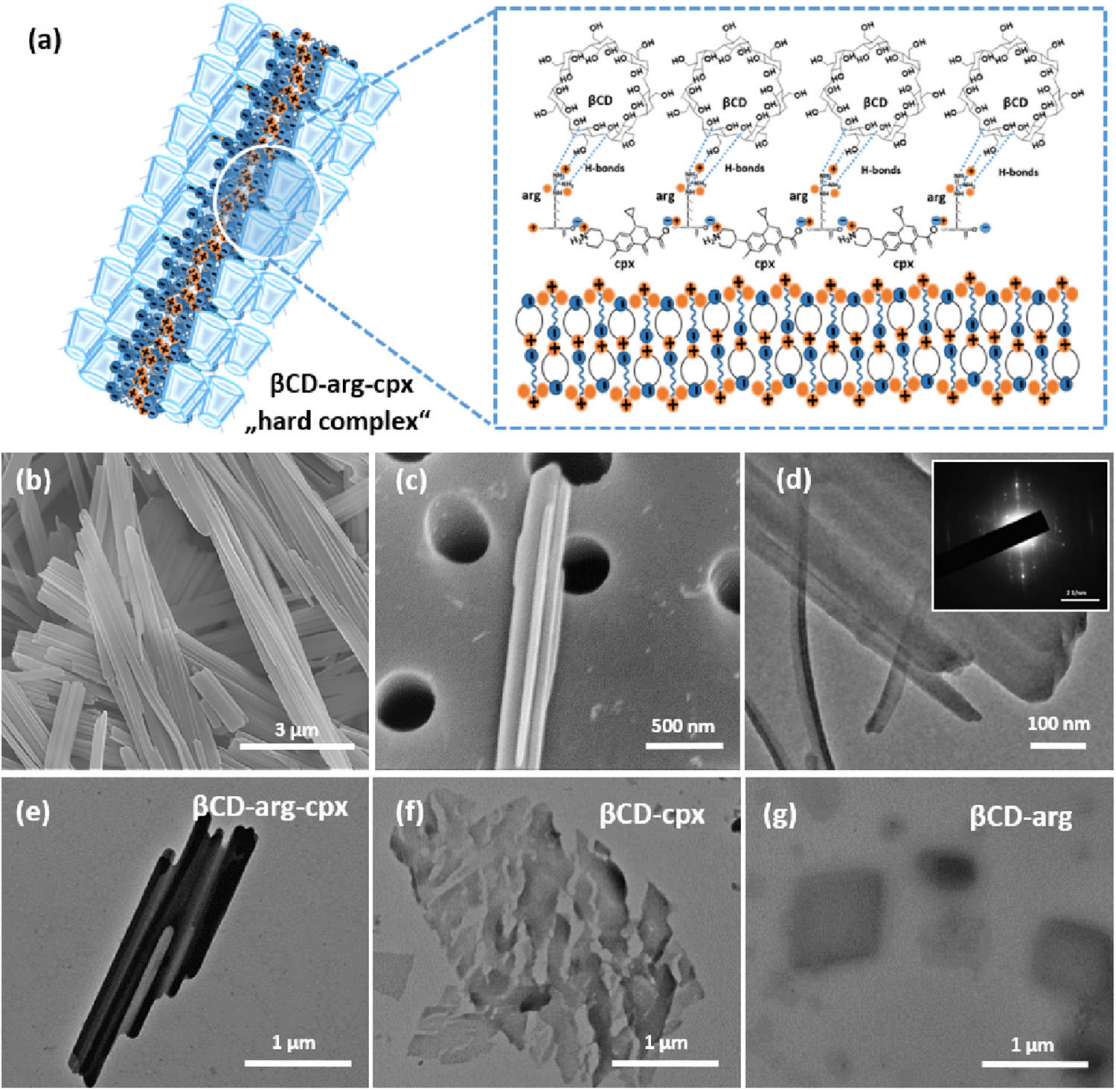
Structural properties of more stable βCD-arg-cpx complex. Illustration of the structure with an arg-cpx crystalline core and βCD at the surface (**a**). SEM morphology showing long, aligned, rod-like structures of βCD-arg-cpx complex (**b**) with sharp, nanothick edges (**c**). Higher-magnification TEM image of the βCD-arg-cpx rods (**d**) and EDS pattern showing their crystalline nature (insert in (**d**)). TEM structures revealing differences among βCD-arg-cpx (**e**), βCD-cpx (**f**) and βCD-arg (**g**).

The βCD-arg-cpx complex assembles into well-organized 3D structures (Fig. 1a) as a few micrometers long rods (Fig. 1b) with highly ordered, radial structures composed of laterally connected plates a few tens of nanometers thick (Fig. 1c). A closer look at the single plates and the associated electron diffraction pattern (Fig. 1d) reveals their single-crystalline nature. Morphologically, these assemblies differ from βCD-cpx, and βCD-arg complexes were detected as irregularly shaped, non-assembled particles (Fig. 1e–g).

**Fig. 2:**
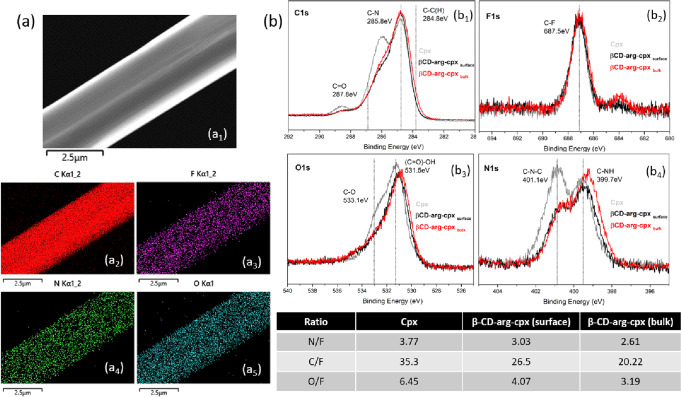
Chemistry of the βCD-arg-cpx stable complex structure. STEM image (a_1_) with elemental mapping analysis corresponding to carbon (C) (a_2_), oxygen (O) (a_3_), nitrogen (N) (a_4_) and fluorine (F) (a_5_); XPS analysis of the surface and bulk (obtained after soft etching) of the βCD-arg-cpx complex in comparison to pristine cpx reference- C1s (b_1_), F1s (b_2_), O1s (b_3_) and N1s (b_4_) high resolution spectra.

Elemental mapping in a single rod βCD-arg-cpx complex (Fig. 2a_1_) detected C (Fig. 2a_2_), F (Fig. 2a_3_), N (Fig. 2a_4_) and O (Fig. 2a_5_) elements homogeneously distributed along the large area of a crystal. Further surface composition XPS analysis was performed on cpx drug reference as well as on βCD-arg-cpx complex before and after soft etching with Ar- ions. Since N is present both in antibiotic and arginine (not in CD), while F is present only in cpx, higher N/F ratio in a complex compare to cpx was due to arginine bonding. On the other hand, decreasing C/F and O/F ratios from surface to bulk of the βCD-arg-cpx complex confirmed βCD at the surface. As F- side of cpx is not included in complexation, the maximum at 687.5 eV in F1s spectrum (corresponding to C-F bonds)^20^ remains unchanged for all three investigated systems (Fig. 2b_2_). On the other hand, N1s (with two maxima at 401.1 eV and 399.7 eV, corresponding to C-N-C and C-NH bonds^20^, Fig. 2b_4_) reveals increased fraction of primarily amines in complex then in cpx free drug which is due to bonding of arginine part. Their increase is observed in bulk area of the complex. The C1s spectrum (with maxima at 287 eV, 285.8 eV and 284.8 eV corresponding to C=O, C-N and C-C, respectively)^20^ and O1s (with maxima at 533.1 eV and 531.5 eV, corresponding to C-O and (C=O)-OH, respectively)^20^) show decrease of intensities of maxima belonging to C-O and C-N groups in a complex compare to the free cpx reference which is due to their involvement in complexation.

The high-aspect-ratio βCD-arg-cpx complex assemblies are highly crystalline, as indicated by their sharp polycrystalline diffraction maxima (Supplementary Fig. 1a). The detected crystal structure is similar to that of the basic deprotonated cpx (as observed in ref. ^21^), with shifted diffraction maxima and the appearance of new peaks. It also differs slightly from the structure detected for the βCD-NaOH-cpx complex (a reference precipitated by replacing arg with NaOH) and exactly matches the structure of arg-cpx (a reference corresponding to the ciprofloxacin-arginine salt) (Supplementary Fig. 1a), which results from the acid-basic reaction between cpx and arg, formation of the salt and its crystallization. In contrast, crystallization of the separated cpx and arg components within βCD-cpx and βCD-arg complex aggregates is low, giving low intensity and broad diffraction maxima (Supplementary Fig. 1a). Due to the absence of the assembly, the crystal order in these structures is notably decreased. Therefore, the crystalline part of βCD-arg-cpx consisted of arg-cpx salt with an amorphous βCD component associated at the crystal surface.

Large structural differences among the different types of complexes were observable particularly in their FTIR spectra (Supplementary Fig. 1b). The FTIR spectrum of the inclusion βCD-arg complex (Supplementary Fig. 1b) showed typical βCD bands with broad O-H stretching at 3300 cm^−1^ and O-H bending at 1640 cm^−1^, and C-H stretching at 2925 cm^−1^, and ring vibration bands as asymmetrical C-O-C and C-C stretching at 1152 cm^−1^, 1026 cm^−1^, and 932 cm^−1^ ^17,22,23^, and guanidine C-N stretching modes from arginine at 1554 cm^−1^ (more details in Supplementary Fig. 2)^24^. The intensity of the O-H stretching and bending modes from βCD was altered in the βCD-arg spectrum, but no additional bands were observed (Supplementary Fig. 2). In the βCD-cpx complex, slight position shifts were observed for the bands corresponding to aromatic ring vibrations of cpx and βCD obtained due to the incorporation of the molecule inside the βCD cone, which is typical of inclusion complexes (Supplementary Fig. 3)^25,26^. In addition, the carboxyl C=O vibrational band of cpx at 1708 cm^−1^ indicated a neutral molecular form^25,26^. The βCD-cpx complex did not show any additional bands (Supplementary Fig. 3).

A completely different situation was observed for the βCD-arg-cpx complex (Supplementary Fig. 1b). The stretching carbonyl C=O vibration mode, detected at 1708 cm^−1^ in βCD-cpx and also in physical mixture of βCD, arg and cpx components, is missing in spectra of both complexes, βCD-arg-cpx and βCD-NaOH-cpx, while complexes’ asymmetric carboxylate vibration (which was missing in case of physical mixture and βCD-cpx) appears at 1578 cm^−1^ both indicating zwitterion cpx forms within the βCD-arg-cpx and βCD-NaOH-cpx (Supplementary Figs. 3 and S4). In contrast to the spectrum of the inclusion complex βCD-cpx and the spectrum of a physical mixture of components from the complex, in βCD-arg-cpx, the cpx O-H stretching vibration at 3526 cm^−1^ was missing, another indication of its deprotonated form within the complex. Moreover, typical vibrational bands observed in previous complexes appeared in the spectrum of βCD-arg-cpx with higher intensity, changed intensity ratios, and higher resolution, indicating an increase in the structural order and crystallinity (as observed to carbonyl group vibrations in formulations containing semicrystalline cpx)^26^. Novel vibrational bands were detected, particularly in the fingerprint area (Supplementary Fig. 4), that did not belong to any of the separate components of the complex (separately and in their physical mixture) but were a consequence of the new complex structure. It was interesting to observe that most of the new bands obtained for βCD-arg-cpx were also detected in arg-cpx and βCD-NaOH-cpx, adjusted to the same pH by using arginine or NaOH, which additionally confirmed the similar structure of complexes formed using these two acidity modulators. However, the presence of arginine within βCD-arg-cpx affected the βCD cycle ring vibrations, observed as the absence of the βCD-typical vibrations at 1079 and 996 cm^−1^ and additional vibration freedom detected through the new band at 1178 cm^−1^, which was not detected in βCD-NaOH-cpx. These new interactions within βCD-arg-cpx could be a source of better complex stability, which will be shown later. Investigations of the optical properties of the βCD- complexes enabled further insights into their structural characteristics. The fluorescence emission spectra of the βCD-arg-cpx and arg-cpx complexes (Supplementary Fig. 1c) showed a bathochromic shift when the βCD component was bonded to arg-cpx salt. A shift was observed in both cases when βCD was bonded to the βCD-arg-cpx or βCD-NaOH-cpx complex (Supplementary Fig. 1c). Indirectly, this observation confirmed the presence of this amorphous component on top of deprotonated cpx crystals (detected in XRD and further revealed in FTIR) and provided evidence of the bonding position. The observed fluorescence was assigned to the antibiotic molecule, with piperazinyl electron donor and 4-oxoquinoline-3-carboxylic acid electron acceptor groups^27^. Similarly, in the present case, cpx is bonded via arg to βCD via a piperazinyl group with an electron donor nature. Bonding the cpx molecule over the arginine linker to βCD (or directly to the βCD molecule) limits its intramolecular motions and eliminates nonradiant relaxations, enabling fluorescence at the λ_max_ shifted compare to free drug form.

**Fig. 3:**
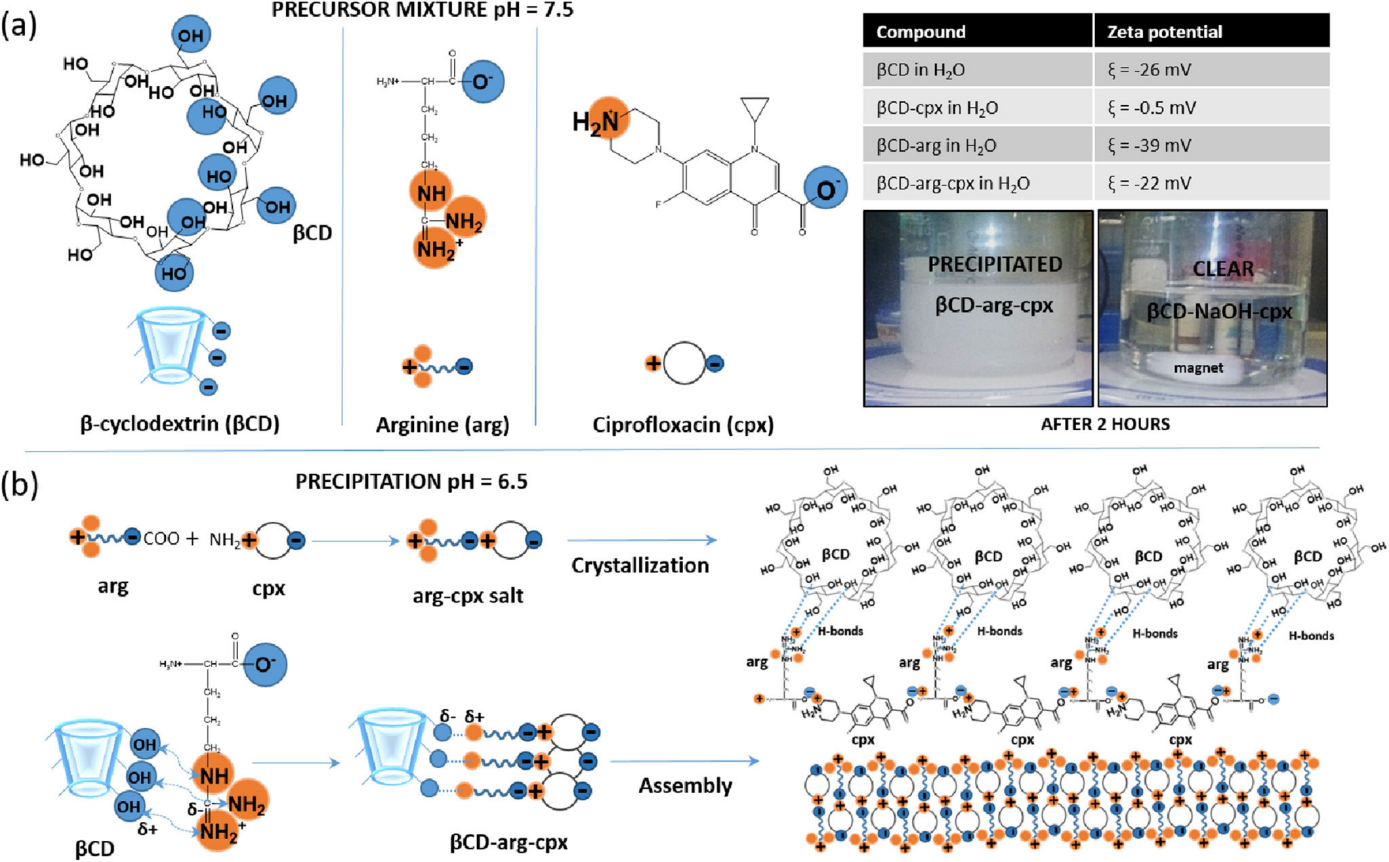
Formation of more stable complex between cyclodextrin (βCD), ciprofloxacin (cpx) and arginine (arg) (βCD-arg-cpx). Molecular structures of the precursor mixture before precipitation (pH 7.5), zeta potential change for different complex components and precipitation after 2 h of mixing in the case of pH adjustment with arginine (and its delay when arg is replaced with NaOH (βCD-NaOH-cpx)) (**a**). Precipitation (pH 6.5) involving arg-cpx salt formation and βCD bonding followed by crystallization and assembly (**b**).

Monitoring pH and zeta potential and replacing arg with NaOH as a pH modifier revealed critical steps toward complex formation (Fig. 3). Initially, the reaction mixture was pH 7.5, at which βCD has available OH groups, arg is in zwitterion form with a cationic charge at guanidine and deprotonated carboxyl groups, and cpx has a cationic amine on piperazinyl and deprotonated carboxyl groups (as illustrated in Fig. 3a). βCD-arg-Cpx complex formation starts with an acid-based reaction in which the carboxyl groups in arg interact with the cationic amine on the piperazinyl cpx side, forming an arg-cpx salt (Fig. 3b). The precipitation crystallization step enables the formation of long rod structures. The surface of the formed crystals has free guanidine groups available for bonding amorphous βCD components and assembling the final CD-arg-Cpx complex (Fig. 3b). Amines in the guanidine functional group in arginine have a very high affinity for hydrogen bonding, with the possibility of forming up to five bonds involving both protonated and nonprotonated amines^28^. Therefore, in the formed complex, arginine has the role of a linker, which uses cationic guanidine groups to bond βCD to the surface and ionic carboxyl groups to bond cpx. The zeta potential of βCD (initially at pH = 6) is changed after adding arginine (Table in Fig. 3), indicating interactions between cationic guanidine backbone groups and OH groups at the hydrophilic outer side of βCD.

The formed CD-arg-cpx complex structure appears as a shiny opal-white precipitate within two hours after mixing all the components. When βCD is mixed directly with cpx without adding arginine, the solution remains clear without any precipitation. Measuring the zeta potential of the lyophilized powder gives neutral values corresponding to nonstable βCD-cpx complex aggregates. As observed earlier, the βCD-cpx inclusion complex forms by incorporating hydrophobic 4-oxoquinoline-3-carboxylic acid moiety of the cpx molecule inside the hydrophobic cone of βCD^29^. When the acidity of the initial reaction mixture was adjusted by using NaOH (instead of arginine), the precipitation of βCD-NaOH-cpx as an arg-free complex was much slower and did not take place after two hours of mixing, as in the case of βCD-arg-cpx (illustrated in photos in Fig. 3a), but was delayed during the following 24 h. βCD-NaOH-cpx is a pH-adjusted βCD-cpx complex and lacks the stability observed for βCD-arg-cpx.

### Detectable antibiotic in the βCD-cpx complexes

**Fig. 4:**
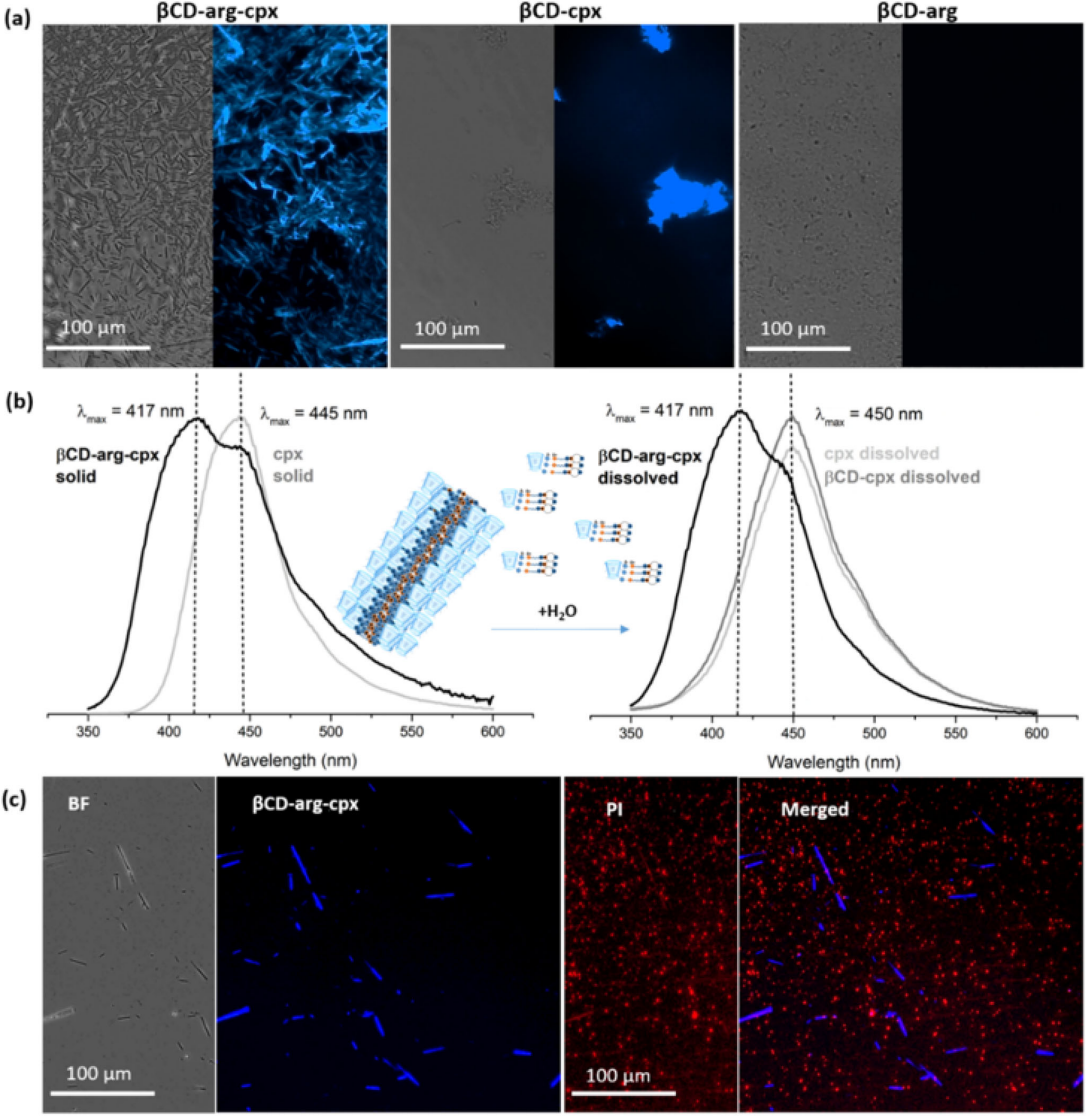
Fluorescence and detection properties of the βCD-arg-Cpx complex. Blue fluorescence of cpx-containing (βCD-arg-cpx and βCD-cpx) complexes (not detected for βCD-arg) (**a**). Fluorescence spectra of βCD-arg-cpx and free cpx (and comparable βCD-cpx complex) before and after dissolving in water confirming different dissolving mechanisms (dissolved complex vs. dissolved free drug) (**b**). Detection of βCD-arg-cpx and of bacteria (100 μg/ml of the complex after 10 min exposure to *P. aeruginosa* PAO1 stained with propidium iodide (PI, red fluorescence, detecting dead bacteria) (**c**).

The βCD-arg-cpx assemblies emitted intense blue fluorescent light that appeared along with their rod-like structures (Fig. 4a). Similar fluorescence was also observed for βCD-cpx (following the irregularly shaped structure of the complex aggregate) (Fig. 4b) but not for βCD-arg (which also forms irregular aggregates) (Fig. 4c). Ciprofloxacin derivatives, in the form of spherical nanoaggregates containing perfluoroaryl and phenyl rings linked by amidine bonds to the piperazinyl group of ciprofloxacin, have been previously observed to show aggregation-induced emission (AIE)^27^. However, to the best of our knowledge AIE in βCD-containing ciprofloxacin complexes has not been detected before.

Differences were also observed in the fluorescent spectra of βCD-arg-cpx and βCD-cpx complexes, particularly before and after dissolving in water (Fig. 4b). In solid powders, the fluorescence spectra of βCD-arg-cpx and free cpx were not the same, revealing a blueshift in the spectrum of the arginine-complexed structure. When dissolved in water, the spectrum of βCD-arg-cpx remained the same as that of the solid complex form, while the spectra of the free drug and less stable βCD-cpx inclusion complex were red-shifted with exactly the same shape of fluorescence maximum. Further analysis of the components dissolved in water revealed free cpx (and its dimers and trimers identified in MS spectra, Supplementary Fig. 5a), while in case of the βCD-arg-Cpx, during aging in aqueous environment the complex released free cpx, cpx-arg, arg and βCD (Supplementary Fig. 5b).

Intensive blue fluorescence provides βCD-arg-cpx with additional detection functionality. After exposure to the βCD-arg-cpx complex for a short time, the viability of bacteria was stongly affected, as detected by the red fluorescence of propidium iodide in a high fraction of cells, which represent dead bacteria. We were also able to detect blue fluorescence from the βCD-arg-cpx rod-like assemblies, which released the complex with strong antimicrobial activity (Fig. 4c).

### Complex dissolution and release

**Fig. 5:**
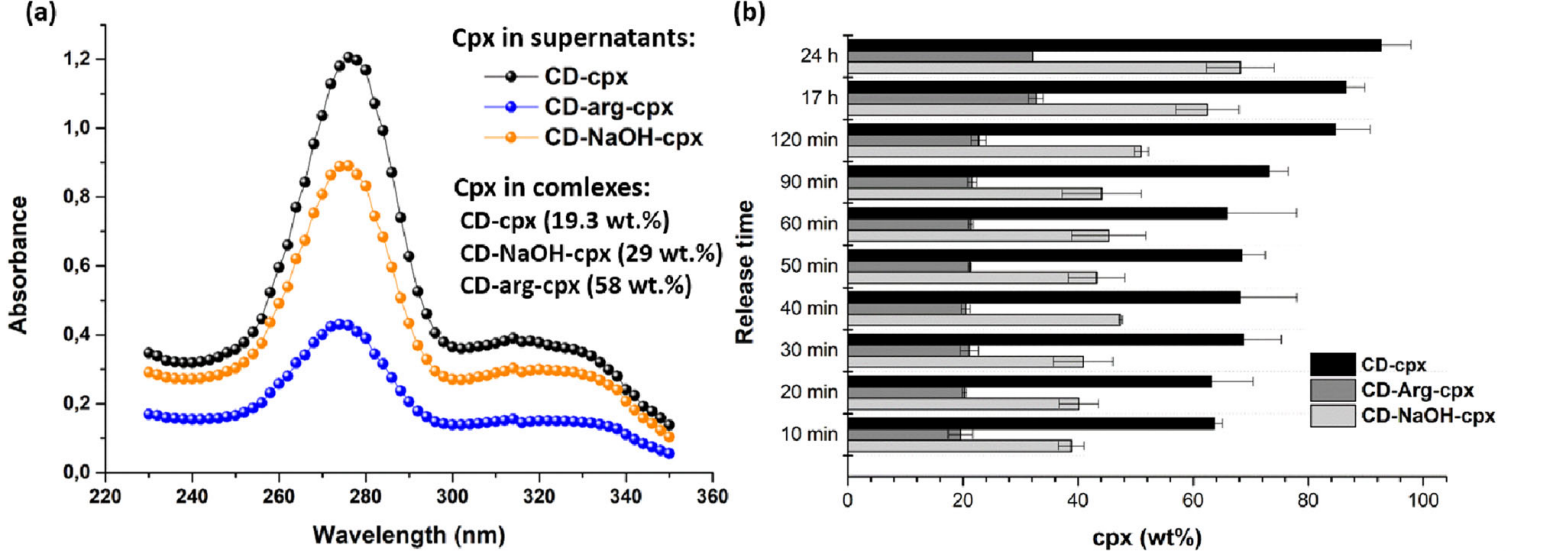
Ciprofloxacin (cpx) contents in complexes and its release. Cpx in supernatants obtained after complex synthesis (and calculated contents of cpx in βCD complexes) (drug released from CD-arg-Cpx complex indicated with blue, from CD-NaOH-Cpx complex in orange and from CD-Cpx in black) (**a**) and comparative drug release kinetics from βCD-cpx (black), βCD-arg-cpx (dark grey) and βCD-NaOH-cpx (lith grey) (**b**); *n* = 3; error bars refer to SD of drug release.

Even in a quantitative context, βCD-arg-cpx differed from the classical βCD-cpx complex (Fig. 5a). As determined from the UV–vis spectra of the remaining cpx in the solution after complex formation, βCD-arg-cpx contained three times the cpx content of βCD-cpx without arginine. The presence of arginine and its role in complex formation affected the drug content within the complex. A 1.5-fold difference in cpx content for βCD-cpx and βCD-NaOH-cpx (Fig. 5b) was observed, showing that a difference in pH did affect the content of the complex-included drug. However, in the case of βCD-arg-cpx, the effect was not solely from the contribution of pH during complex formation, since using arginine to modify the pH to the same level as for NaOH within the βCD-NaOH-cpx complex resulted in a two times higher cpx content in βCD-arg-cpx.

Along with its contribution to the formation, assembly, and incorporated drug content, arginine affected the release of the drug from the complex. In a classical inclusion βCD-cpx complex, a less stable dynamic balance between the drug and the βCD hydrophobic cone, cpx release is fast (Fig. 5b). After incubation at 37 °C in PBS buffer, more than 60% of the drug was released after 10 min of incubation. The complete decomposition of the complex and release of all the incorporated cpx took place within the first 24 h. The release kinetics were slightly slower in the case of βCD-NaOH-cpx (Fig. 5b): the initial release was approximately 40%, and at the end of 24 h, the complex released up to 70% of the incorporated drug.

In drug delivery systems (like encapsulated polymeric spheres) drug release depends on properties of the drug carrier (i.e. polymer matrix degradation, swelling, T- /pH - dependent response) which enables physical release or desorption of the entrapped drug. In current case the drug, as part of the complex, is a part of drug delivery system and its release depends on stability and solubility of the complex. Due to the difference in the assembly the βCD-arg-cpx complex was more stable than the inclusion complex of βCD-cpx and the pH-modified βCD-NaOH-cpx complex, showing the slowest release (Fig. 5b). Self-assembly of the natural CDs and drug complexes is known to affect their solubility^12^. Initially, this complex released only 20% of the incorporated drug, and the release continued slowly, with up to 30% of the drug released after 24 h. The application of βCD as an excipient or the application of arginine to create a drug salt within βCD-drug complexes is a convenient way to enhance drug solubility. In contrast, the novel βCD-arg-cpx complex disassembles slowly, decreases drug dissolution and enables control over drug release. The observed contribution to drug solubility is a consequence of the different bonding of the drug to the other two components (as illustrated in Fig. 3). Instead of incorporating the hydrophobic part of the drug within the βCD cone and bonding arginine to its hydrophilic part, which usually remains outside the βCD cone, the drug is bonded to the βCD surface via an arginine linker, which makes the whole structure more stable against dissolution. Moreover, the forces that hold together the long rod-like assemblies of βCD-arg-cpx also contribute to slower disassembly and provide release control. In contrast to all of the available CD-associated platforms used to modify antibiotics, to the best of our knowledge the possibility of using arginine in creating a slowly-release antibiotic drug delivery system from βCD has not been conferred before.

### In vitro antimicrobial efficacy and safety

Having observed the unconventional decreased solubility and slowed antibiotic release from the βCD-arg-cpx assembly, we next investigated of the effect of the complex in antimicrobial efficacy and toxicity testing. First, the susceptibility and minimal inhibitory concentrations (MICs) of βCD-arg-cpx and βCD-cpx complexes and their precursor components, βCD, βCD-arg, and cpx, were investigated against several bacterial strains, including gram-negative *E. coli* MG1655 (ATCC 47076) and *P. aeruginosa* PAO1 (ATCC 15692) and gram-positive *S. aureus* Rosenbach (ATCC 12600), as summarized in Table 1. None of the investigated bacterial strains showed any susceptibility to βCD or βCD-arg. For βCD-cpx, we found MIC values of 0.056 μM for *E. coli* and 0.21 μM for *P. aeruginosa* and *S. aureus* (Table 1). If we take into account the 19-wt% cpx content in βCD-cpx and up to 70% drug release during a 24-h incubation at 37 °C (Fig. 5b), the MICs correspond to 0.0074 μM and 0.028 μM free drug equivalents. Relative to the cpx reference, the βCD-cpx inclusion complex provided increases of approximately 28-fold in antimicrobial activity for all bacterial strains tested. The antibacterial efficacy of βCD-arg-cpx was higher than those of the others with MICs of 0.0045 μM for *E. coli*, 0.018 μM for *P. aeruginosa* and 0.060 μM for *S. aureus*. With 58 wt% cpx content and up to 30% drug release (Fig. 5b), the MIC was equal to 0.0008 μM, 0.003 μM and 0.010 μM free cpx equivalents, respectively. Accordingly, compared to the cpx reference, βCD-arg-cpx had 260-fold, 260-fold and 78-fold increased antimicrobial activity for the bacterial strains tested, respectively.

**Table 1:** Minimal inhibitory concentrations (MICs) of βCD and its complexes against gram-positive and gram-negative bacterial strains, their cytotoxicity to mammalian cells and their selectivity indices.

*E. coli*	*P. aeruginosa*	*S. aureus*	CC_50_ (μM)
NA/NA	NA/NA	NA/NA	NT
NA/NA	NA/NA	NA/NA	NT
0.208/0.052 (59,615)	0.780/0.520 (5962)	0.780/0.520 (5962)	>3100
0.056/0.014 (97,143)	0.210/0.140 (9714)	0.210/0.140 (9714)	>1360
0.0045/< 0.0015 (813,333)	0.018/0.012 (101,666)	0.060/0.0049 (248,979)	1220

Minimal inhibitory concentrations (MIC) and 50% inhibitory concentrations (IC_50_) for *Escherichia coli* (*E. coli*), *Pseudomonas aeruginosa (P. aeruginosa)*, and *Staphylococcus aureus* (*S. aureus*). Cytotoxic concentrations (CC_50_) were evaluated in lung epithelial A549 cells. The selectivity index (SI), calculated as CC_50_/IC_50_, is indicated in parentheses next to the IC_50_. β-Cyclodextrin (βCD), 2-β-cyclodextrin-arginine (βCD-arg), 3-ciprofloxacin (cpx), 4-β-cyclodextrin-ciprofloxacin (βCD-cpx), and 5- β-cyclodextrin-arginine-ciprofloxacin (βCD-arg-cpx).

*NA* no detected activity, *NT* noncytotoxic.

**Fig. 6:**
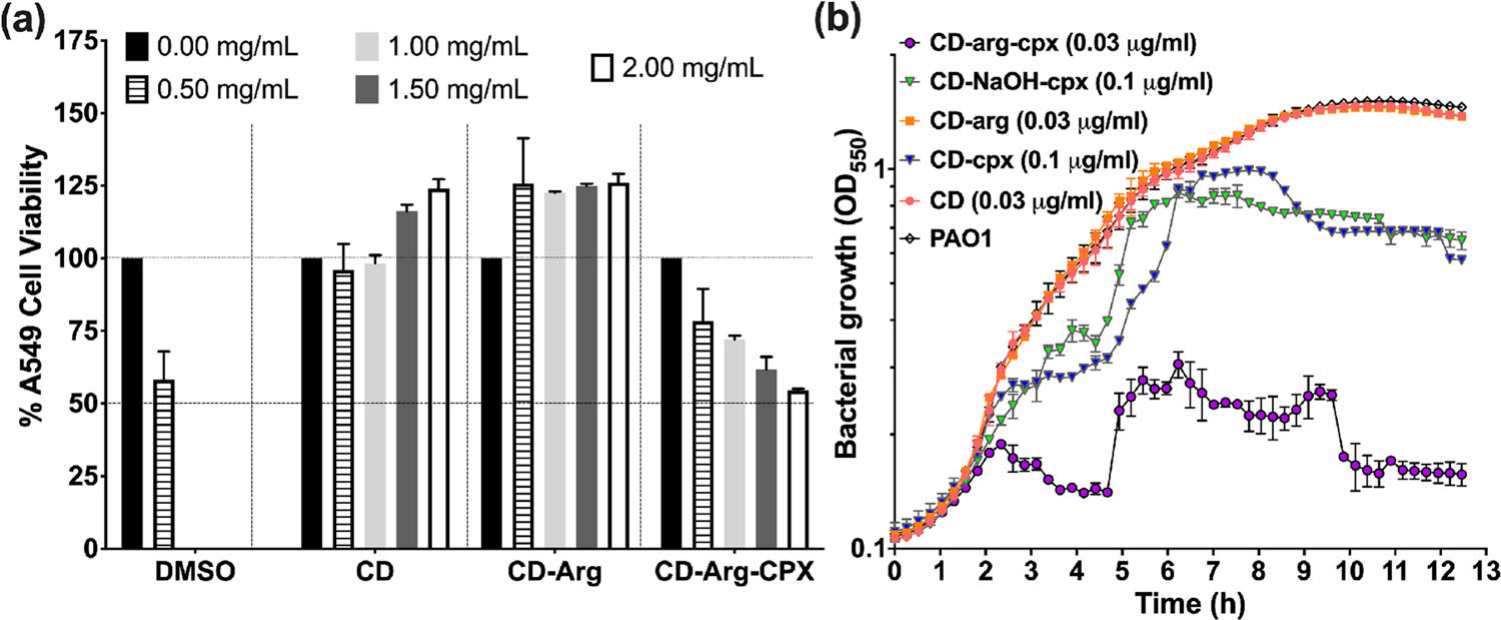
Antimicrobial properties vs. cytotoxicity of CD complexes. Cytotoxicity of βCD-arg-cpx and its components to lung epithelial A549 cells (**a**). The plot shows the percentage of A549 cell viability after incubation with different concentrations of βCD complexes (0 mg/ml (black), 0.5 mg/ml (patterned), 1 mg/ml (light grey), 1.5 mg/ml (dark grey) and 2 mg/ml (black border). DMSO was used as a positive control for toxicity, while untreated A549 monolayers were used as a negative control for cytotoxicity; *n* = 3; error bars refer to SD of viability relative to non-treated cells. Comparison of *P. aeruginosa* PAO1 growth (white) in the presence of βCD-arg-cpx (0.03 μg/ml or 0.020 μM) (purple), βCD-NaOH-cpx (0.2 μg/ml or 0.132 μM) (green), βCD-cpx (0.2 μg/ml or 0.140 μM) (blue), βCD-arg (0.03 μg/ml or 0.0023 μM) (orange) and βCD (0.03 μg/ml or 0.0026 μM) (rose) (**b**); *n* = 3; error bars refer to optical density SD.

Cytotoxicity in mammalian cells was evaluated using lung epithelial cells (A549) (Table 1 and Fig. 6a). The βCD-arg-cpx complex was characterized as nontoxic (with a high CC_50_ value of 2000 μg/ml (1220 μM)). Only high-concentration doses (>1000 μg/ml (610 μM)) of βCD-arg-cpx showed a dose-dependent reduction in cell viability (Fig. 6b), which corresponded to approximately 100,000–800,000 times higher MICs. The complexation of cpx to βCD decreases the toxicity (Table 1). Furthermore, it is important to note that the selectivity index (SI) increased in the different bacterial tests compared to that of soluble cpx (13 times for *E. coli*, 17 times for *P. aeruginosa*, and 42 times for *S. aureus*) (Table 1). In other words, while nanomolar concentrations exhibited efficient antimicrobial activity, millimolar concentrations were needed for toxic effects, presenting an extensive therapeutic window for safe application of the complex and increasing the possible in vivo efficacy compared to soluble commercial cpx.

We next investigated the antimicrobial activity by analyzing MIC values and kinetics in bacterial growth, as presented in Fig. 6b for the case of *P. aeruginosa*. More details on microdilution test performed for βCD-arg-cpx and βCD-cpx complexes, CD-arg and CD auxiliary components as well as direct comparison of the βCD-arg-cpx to free cpx drug are presented in Supplementary Fig. 6. The complex components, βCD, and βCD-arg did not alter bacterial growth. On the other hand, at 0.2 μg/ml, both βCD-cpx and βCD-NaOH-cpx (as βCD-cpx version obtained in alkaline medium) (with 0.140 μM and 0.132 μM molar concentrations, respectively) slowed bacterial growth with clear bacteriostatic effects, while βCD-arg-cpx enabled bactericidal effects at 0.03 μg/ml (0.018 μM). These results provided clear evidence that the improvement in antimicrobial activity for βCD-arg-cpx is not a consequence of the pH at which the complex is formed (although βCD-NaOH and βCD-arg-cpx have similar structures). Moreover, it directly reflects the special role of arginine as a linker. The decrease in MIC between the classical less stable βCD-cpx inclusion complex and more stableβCD-arg-cpx assembly may be a consequence of how they release and disassemble. Due to weak interactions between antibiotics and βCD in the inclusion complex, when they dissolve, the complex decomposes, enabling the release of the free drug. On the other hand, when arginine connects βCD and cpx, the binding forces are stronger, and disassembling the rod-like structures results in a gradual release of free cpx and also cpx-arg as more active drug form.

**Fig. 7:**
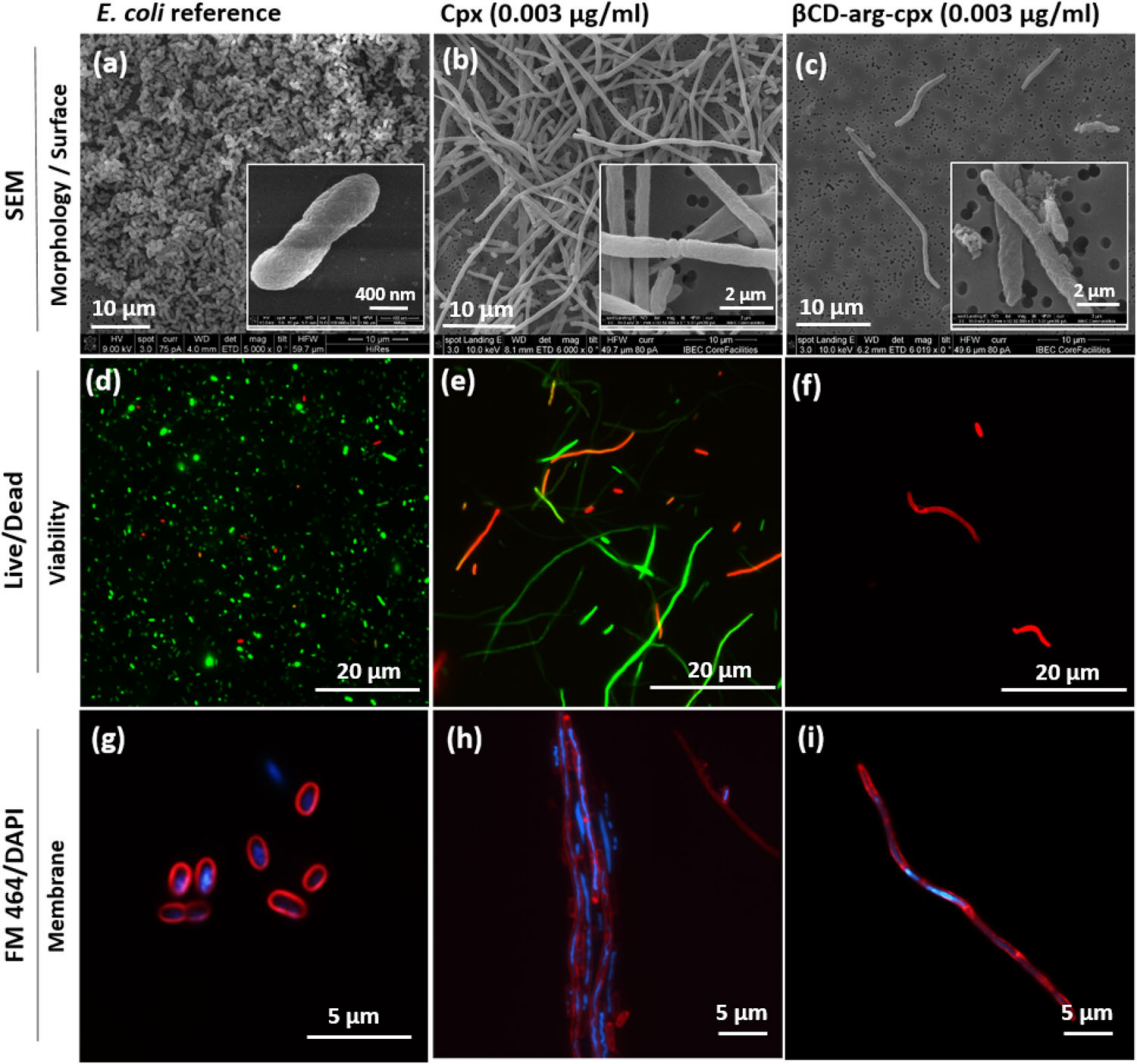
*E. coli* MG1655 exposed to ciprofloxacin and the βCD-arg-cpx complex. Morphology and surface of *E. coli* cells before (**a**) and after exposure to low cpx concentration (0.003 μg/ml or 0.008 μM) (**b**) and equivalent of cpx in βCD-arg-cpx (0.003 μg/ml or 0.002 μM) (**c**); Live *E. coli* stained with a Live/Dead dyes (**d**) and fraction of viable *E. coli* cells treated with 0.003 μg/ml (0.008 μM) cpx (**e**) and βCD-arg-cpx (0.002 μM) (**f**). Membrane structure of wild-type *E. coli* stained with FM464/DAPI dyes (**g**) and bacteria treated with 0.003 μg/ml pure cpx (0.008 μM) (**h**) and CD-arg-cpx complex (0.002 μM) (**i**).

**Fig. 8:**
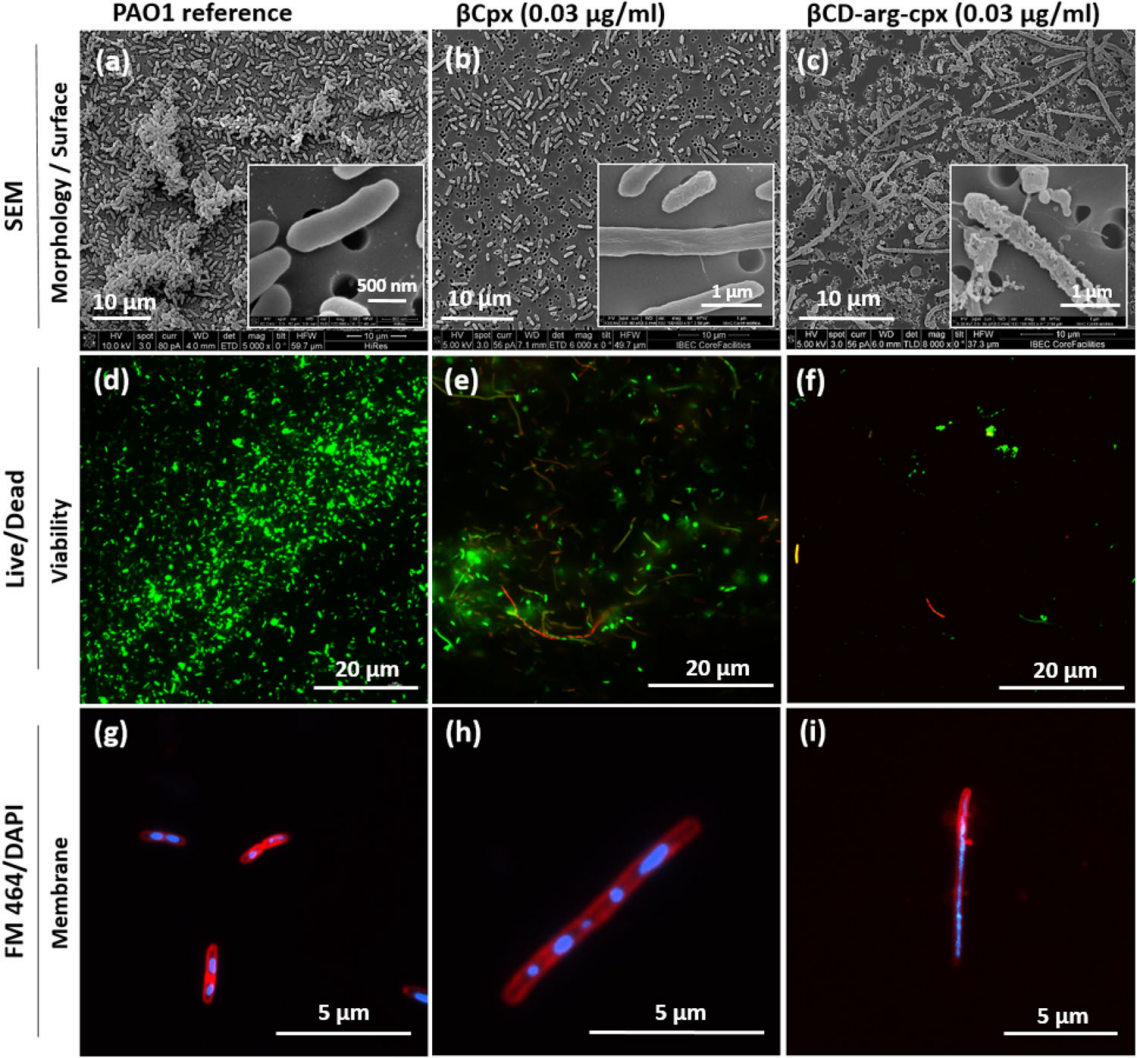
*P. aeruginosa* PAO1 exposed to ciprofloxacin and its βCD-arg complex. Morphology and surface of PAO1 cells before (**a**) and after exposure to low cpx concentration (0.03 μg/ml or 0.08 μM) (**b**) and equivalent cpx in βCD-arg-cpx (0.03 μg/ml or 0.02 μM) (**c**). Live *P. aeruginosa* PAO1 stained with a Live/Dead dyes (**d**) and fraction of viable cells treated with 0.03 μg/ml cpx (0.08 μM) (**e**) and βCD-arg-cpx (0.02 μM) (**f**). Membrane structure of wild-type *P. aeruginosa* PAO1 stained with FM464/DAPI dyes (**g**) and bacteria treated with 0.03 μg/ml pure cpx (0.08 μM) (**h**) and βCD-arg-cpx complex (0.02 μM) (**i**).

In a morphological context, after cpx and βCD-arg-cpx treatment of gram-negative bacteria (*E. coli* and *P. aeruginosa*), we observed bacterial filamentation (Figs. 7 and 8, respectively). This was an evidence that the primary antibacterial mechanism of cpx was not changed, and it was also observed when the drug was within the βCD-arg-cpx complex assembly. Cpx is well known to affect DNA replication by targeting DNA gyrase, which inhibits bacterial cell division^30,31^. Under antibiotic-induced stress, bacteria that stop cell division continue their growth by inhibiting cell septation, increasing their length, and forming long filaments^32^. Depending on the stress conditions, bacteria inside filaments can survive or die^30^. If individual cell units connected within a filament remain viable and form long multicellular chains, stress relief occurs in the evolving antibiotic-resistant strain^31^. In that context, subinhibitory concentrations of cpx induce the filamentation of rod-shaped bacteria as a transition step toward their evolution into cpx-resistant strains^32^. After treatment with very low, subinhibitory cpx concentrations, 0.003 μg/ml (0.008 μM) in *E. coli* and 0.03 μg/ml (0.08 μM) in *P. aeruginosa*, we observed very intensive and long filamentation in *E. coli* (Fig. 7b, e, h) (compare to non-treated reference (Fig. 7a, d, g), while *P. aeruginosa* showed only cell elongation with few cases of very long filaments (Fig. 8b, e, h) (which was not detected in reference (Fig. 8a, d, g)). Connected division septa, observed in SEM images, resulted from bacterial inhibition of cell division. Only some filamented cells were dead, as revealed by live/dead staining, while the majority remained alive and survived the stress induced by the low concentration of cpx (Figs. 7e,  8e). The bacterial cell membrane remained intact (as shown by staining with FM464 only at the surface) and clearly showed several nucleosomes along the filaments (stained with DAPI) (Figs. 7h,  8h). We also observed normal-sized bacteria connected to the ends of the filaments (Fig. 7h), indicating debugging and the possible formation of antibiotic-resistant strains.

A completely different situation was seen when treating at the same concentrations of the βCD-arg-cpx complex used for soluble cpx (Figs. 7, 8). In *E. coli*, a very low cpx concentration (0.003 μg/ml (0.002 μM) of the βCD-arg-cpx complex effectively stopped bacterial growth and decreased the number of filamented cells (Fig. 7c). The filaments contained intact cell membranes and multinucleosome entities (Fig. 7i). Most importantly, all of the detected bacteria, normal-sized and filamented, were confirmed to be dead (Fig. 7f). A similar result was obtained for *P. aeruginosa* exposed to a low cpx concentration (0.03 μg/ml (0.02 μM)) of the βCD-arg-cpx complex. Although the number of formed filaments increased, a closer look at their surface revealed strong damage, while their environment showed many cell fragments remaining after decomposition (Fig. 8c). Similar decomposition of *P. aeruginosa* was detected after exposure to a high cpx dose (0.2 μg/ml (0.52 μM), which resulted in increased membrane vesicle formation induced by an explosive cell lysis mechanism^30^. As observed in *E. coli*, all the filamented *P. aeruginosa* were dead (Fig. 8f). FM646/DAPI revealed their multinuclear structure and membrane deformation into vesicles (Fig. 8i). Deformation of the membrane, vesicle formation, and cellular damage by explosive cellular lysis were progressive and depended on the βCD-arg-cpx complex concentration (Supplementary Fig. 7). At lower concentrations (0.03 μg/ml (0.02 μM), both filaments and normal-sized cells were detected. They all had damaged cell walls containing small holes and were partially decomposed into vesicles (200 nm in size). At a higher βCD-arg-cpx concentration (0.1 μg/ml (0.06 μM)), filaments were no longer detected, and all of the remaining normal-sized cells were completely lysed into vesicle-like cell wall fragments approximately 100 nm in size. Progressive cell damage indicated their response to the increasing stress induced by the cpx loaded in the complex. Similar changes including intensive deformations in the membrane, damage to the structure of the cells, and finally antimicrobial activity when the βCD-arg-cpx complex was used at much lower concentrations than the free cpx drug were also observed for *S. aureus*, a gram-positive strain (Supplementary Fig. 8).

### In vivo antimicrobial efficacy and safety

**Fig. 9:**
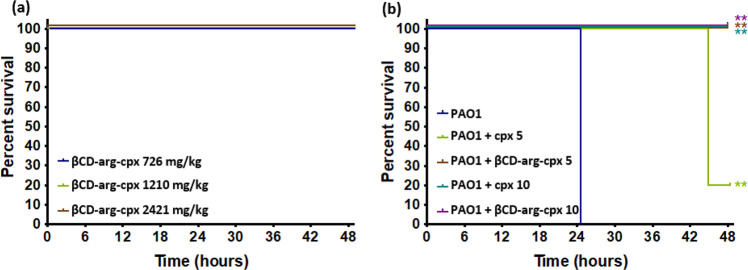
Kaplan–Meier survival curves for in vivo toxicity and efficacy in the *Galleria mellonella* larva model. Toxicity of βCD-arg-cpx complex showing very high larval survival for the whole concentration range tested (up to 2421 mg/kg) (**a**). Comparative antibacterial efficacy study in larvae infected with PAO1 (dark blue) and treated with the βCD-arg-cpx complex (light green and brown) and free cpx drug (dark green and purple) (equivalent cpx content of 5 and 10 mg cpx/kg larva, respectively) that shows higher survival of the preinfected worms after treatment with the complex (**b**). Asterisks: statistically significant differences versus *P. aeruginosa* PAO1 control in a log-rank test (**p value < 0.01); *n* = 5. This figure represents the same experiment repeated several times, which yielded identical results every time.

The in vivo toxicity and efficacy of the βCD-arg-cpx complex were investigated in *Galleria mellonella* worms (Fig. 9) as an animal model previously optimized to evaluate antibacterial efficacy and toxicology^33,34^. Toxicity was investigated at high concentrations of the complex (up to 2421 mg/kg), and 100% survival was observed for all of the investigated concentrations. For the antibacterial efficacy study, worms were preinfected with *P. aeruginosa* PAO1^35^. Without any treatment, 100% death was obtained after 24 h. Postinfection treatment with βCD-arg-cpx at concentrations of 5 mg/kg (3.3 mM) and 10 mg/kg (6.6 M) resulted in 100% survival. As a reference, treatment with 5 mg/ml (13.3 mM) free cpx resulted in only 20% survival. Better efficacy of treatment with the βCD-arg-cpx complex in comparison with the free drug is further supported by the fact that the complex contains only 58 wt% cpx, which is slowly released from the assembled structures (as shown in Fig. 5).

The basic underlying mechanism of antimicrobial action in the assembled βCD-arg-cpx complexed drug remains the same as that of its free form drug. The βCD-arg-cpx complex has the same ability to inhibit cellular division progress typically obtained with free cpx as well as to induce similar stress-related changes in bacteria, such as filamentation and explosive lysis driven by membrane vesicle formation. Although these two drug forms use the same mechanism of action, a difference in their efficacy is evident and could be attributed to a difference in their bioavailability. The presence of the βCD component, with the known ability to increase interactions with cell wall components^12^, certainly promotes drug transfer through the membranes and consequently increases the antimicrobial efficacy at available concentrations inside the bacterial cell. However, as has been seen after direct comparison of less stable βCD-arg inclusion and more stable βCD-arg-cpx complexes, the effect cannot be attributed solely to the presence of βCD. Due to the reversible nature of bonding in inclusion complexes, solubilization provides the free drug form in balance with βCD molecules. This type of balance enhances drug transfer across the membrane but is far less effective as more stable βCD-arg-cpx complex form. More stable βCD-arg-cpx, in which βCD and cpx are bonded via an arginine linker, enables βCD to act as a building component of the drug molecule. Along with βCD, the transfer of the drug through the membrane is also affected by the presence of arginine and its bonding to drug molecule.

Consequently, complexed cpx was more effectively transferred to its target (DNA gyrase) and was able to induce the same level of stress as much higher concentrations of free cpx. If the complex concentrations were high enough (still much lower than the effective concentrations of free drug), the interactions of βCD and arginine bonded to the drug with bacterial wall components were sufficient to disintegrate the bacterial cell structure and induce an explosive lysis process. All these facts indicate that stress induced at very low concentrations of βCD-arg-cpx, enables an important enhancement in antimicrobial activity. Detailed investigations of the potential of the complexed drug form to prevent adaptive evolutionary processes that produce resistant strains remains as interesting challenge for the future.

## Conclusion

This innovative type of βCD complexes, in which linkers are used to make more stable connections between βCD and a drug, hold potential for enhancing the efficacy of previously approved, clinically used antibiotics. Produced using very simple, technologically non-demanding, and highly economical chemistry, such complexes could be very promising tools for repurposing, reprofiling, or reusing different drugs. Future research in this area should be focused on exploring all of the available possibilities introduced by enhancing drug bioavailability within this type of complex, including their capacity to develop and affect antibiotic-resistant strains, mitigate drug toxicity, and enhance biocompatibility. Such complexes can offer additional options in antibiotic pipeline and encourage the pharmaceutical industry to explore more effective forms of old antibiotics and to actively participate in resolving the global lack of effective antibiotics as a persistent and very serious health problem in the modern era.

## Methods

### Materials

The complex was formed using L-arginine (arg, L-2-amino-5-guanidinopentanoic acid, C_16_H_14_N_4_O_2_, Sigma–Aldrich, Germany), ciprofloxacin-hydrochloride (cpx, Cayman Chemical Company) and β-cyclodextrin (βCD, Sigma–Aldrich C4805, Germany). All the chemicals and reagents used were of analytical grade. All of the experiments were performed using laboratory-produced ultradistilled water.

### βCD-arg-cpx complex synthesis

Before forming the complex, arginine (50 ml, 0.4 mg/ml) and cyclodextrin (50 ml, 2 mg/ml) were dissolved in water with continuous stirring (200 rpm) for 15 h at room temperature. The premixed solutions were added to aqueous ciprofloxacin solution (0.8 mg/ml), and stirring (200 rpm) was continued for the next 24 h. The white precipitate appeared 2 h after ciprofloxacin addition. After 24 h of mixing, the supernatant was separated by centrifugation (8000 rpm), and the precipitate was redispersed in 5 ml of distilled water. The dispersion was frozen in dry ice (for 3 h) and freeze-dried (24 h, Christ Alpha 1–4, Martin Christ). Control samples were formed following the same protocol but with replacement of arginine with a water solution of NaOH to form βCD-NaOH-cpx and with replacement of arginine with distilled water to form βCD-cpx. Due to a lack of precipitation in control samples, the total volume of the mixture was frozen and freeze-dried. All powders were kept in covered glass containers and stored under ambient conditions for further testing.

### Physicochemical characterization

Powder X-ray diffraction analyses (pXRD) were performed in the 2–70° 2Θ range with a 0.02° step size and 2 s per step recording using a Bruker AXS D4 Endeavor diffractometer. Fourier transform infrared spectroscopic (FTIR) investigations were performed by a Perkin Elmer Spectrum 400 MIR spectrophotometer using the DRIFT technique. Spectra were recorded in powders obtained by mixing 5 mg of samples with 80 mg of KBr. X-ray photoelectron spectroscopy (XPS) was performed with the Versaprobe 3 AD (Phi, Chanhassen, US) using monochromatic Al-Kα X-ray source. For each measurement, spectra were acquired on a 200 µm spot size with the charge neutralizer turned on, as the pellets were put on a non-conductive double tape. Survey spectra were measured at 280 eV pass energy and step of 1 eV, while high-resolution spectra were measured at 27 eV pass energy and step of 0.025 eV. Charge neutralization was used, so the energy scale of XPS spectra were corrected with shifting C1s peak of carbon to binding energy of 284.8 eV. XPS data were analysed with PHI Multipak software. In order to remove surface layer of the pellet to examine underlying surface, sputtering of the sample with argon clusters was used. Sample was sputtered for 1 min at 5 kV and 20 nA over 2 × 2 mm area, followed by 200 µm high-resolution point analysis. Dissolution products were identified using mass spectrometry. Measurements were performed using a hybrid orthogonal acceleration time-of-flight mass spectrometer Q-Tof PremierTM (Waters-Micromass, Manchester, U.K.) equipped with atmospheric pressure ionization (API) sources and coupled with an ultra-performance liquid chromatograph (TOF MS/UpLC). The detection was performed via an electrospray ionization (ESI) source in positive mode. For morphological scanning electron microscopy (SEM, Nova NanoSEM), powders were dispersed in water and deposited on 50-nm filter membranes stuck on carbon tape and coated with 5 nm carbon. Structural transmission electron microscopy analyses (TEM, Tecnai Spirit 120 kV) were performed on samples dispersed in water and deposited on copper grids. STEM images were taken with Verios 4 G HP (Thermo Fisher Scientific, Waltham, Massachusetts, USA) scanning electron microscope (SEM) in STEM mode. Energy-Dispersive X-ray Spectroscopy (EDXS) system with Ultim Max 65 detector and AZtec software (Oxford Instruments Abingdon, United Kingdom) was used for the EDS mapping. Fluorescence imaging was performed on water-dispersed powders dropped on microscope slides using a Nikon inverted fluorescence microscope ECLIPSE Ti-S/L100 (Nikon) coupled with a DS-Qi2 Nikon camera. Zeta potentials were measured using a Mavern Nanosizer.

### Antimicrobial tests

Antibacterial testing was performed using *Escherichia coli* MG1655 (ATCC 47076), *Pseudomonas aeruginosa* PAO1 (ATCC 15692), and *Staphylococcus aureus* Rosenbach (ATCC 12600). The strains were cultured overnight at 37 °C in liquid growth medium (Luria Bertani, LB) (Scharlab, Spain) for *E. coli* and *P. aeruginosa* and tryptic soy broth (TSB) (Scharlab, Spain) for *S. aureus*. Bacterial samples were ultrasonically dispersed in growth medium for 30 s (A = 18%, W = 250 W, on:off = 2:1 s) to form 2 mg/ml stocks, which were further diluted to the tested concentrations. Microtiter plate wells (96-well assay plate, tissue culture-treated polystyrene; Costar 3595, Corning Inc., Corning, NY) were inoculated with 100 μl of bacteria (OD_550_ = 0.05) and 100 μl of the specific serial dilutions of the tested samples. Controls included growth medium and bacteria without treatment. Incubation was performed in an Infinite M200 Pro multimode microplate reader (Tecan) at 37 °C for 14 h with continuous orbital shaking. Bacterial growth was assessed by measuring optical density at 550 nm every 15 min. All concentrations were tested repeatedly in three replicates (*n* = 3).

### Live/Dead study in bacteria

To assess bacterial viability, the tested samples were dispersed in LB or TSB growth medium for 30 s (A = 18%, W = 250 W, on:off = 2:1 s) and mixed with bacteria to a final volume of 1 ml (OD_550_ = 0.3). After incubation for 12 h, 100 μl was centrifuged for 5 min at 6000 rpm, and the supernatant was replaced with 25 μl of Live/Dead BacLight Bacterial Viability Test (Invitrogen, Thermo Fisher Scientific) containing SYTO9 and propidium iodide (PI) in 1X phosphate-buffered saline (PBS) at a 1:1 ratio and a concentration of 3 × 10^−6^ mg/ml. It was followed by a 15-min incubation in the dark to stain the bacteria. Fluorescent bacteria were visualized by a Nikon inverted fluorescence microscope ECLIPSE Ti-S/L100 (Nikon) coupled with a DS-Qi2 Nikon camera (Nikon).

### FM 464/DAPI study in bacteria

To assess membrane integrity, the tested samples were dispersed in LB or TSB growth medium for 30 s (A = 18%, W = 250 W, on:off = 2:1 s) and mixed with bacteria to a final volume of 1 ml (OD_550_ = 0.3). After incubation for 12 h, 100 μl was centrifuged for 5 min at 6000 rpm, and the supernatant was replaced with 50 μl of FM 464 (N-(3-triethylammonium propyl)-4-(6-(4-(diethylamino)phenyl)hexatrienyl)pyridinium dibromide, Invitrogen, Thermo Fisher Scientific)/DAPI (diamidino-2-phenylindole (DAPI; Biotium, Fremont, CA) in Hank’s balanced salt solution (HBSS) (containing 0.4 μl of 5 mg ml-1 FM 464 and 1 μl of 125x DAPI). Samples were stained for 15 min in ice, light protected, and subsequently analyzed using a Nikon fluorescent ECLIPSE Ti-S/L100 microscope.

### Scanning electron microscopy study in bacteria

Morphological analyses of bacterial cells affected by the investigated complex were performed using FEISEM (Nova NanoSEM). One hundred microliters of bacteria incubated with the complex for 12 h was centrifuged for 5 min at 6000 rpm and fixed after replacing the growth medium with 50 μl of glutaraldehyde (3 wt %). The fixation step was performed for 3 h at room temperature. Fixed bacteria were deposited on porous membranes by filtration under soft vacuum, washed three times with PBS (for 15 min for each wash), and dehydrated in serially diluted ethanol (30, 50, 70, 90, and 100 wt %, 30 min at each concentration). The samples were dried at critical points.

### In vitro toxicity study

Testing was performed in human lung epithelial A549 cells (ATCC® CCL-185™). Cells were cultured in DMEM/F-12 (Gibco, Thermo Fisher Scientific) supplemented with 1% (v/v) penicillin–streptomycin (Gibco, Thermo Fisher Scientific) and 10% (v/v) decomplemented fetal bovine serum (Gibco, Thermo Fisher Scientific) and grown in a humidified incubator (Memmert) at 37 °C and 5% (v/v) CO_2_. The tested complex was ultrasonically dispersed in DMEM for 30 s (A = 18%, W = 250 W, on:off = 2:1 s) to form a stock solution (2 mg/ml). Cells grown to confluence in a 96-well plate were treated with serial dilutions of the complex and incubated at 37 °C in 5% CO_2_ for 24 h. The cytotoxicity assay was performed by adding 20 μl of 10 x Presto blueTM Cell Viability Reagent (Molecular Probes, Invitrogen, Thermo-Fisher Scientific) per well, incubating for 30 min and recording fluorescence at excitation λ = 560 nm and emission λ = 590 nm. The references included the complex without cells in DMEM, the pure dye in DMEM, cells without the complex (as a negative control), and cells with DMSO (as a positive control). All concentrations were tested repeatedly in triplicate (*n* = 3). Viability has been normalized to cells without treatment (viability % to negative control).

### In vivo toxicity

*Galleria mellonella* larvae, used as an in vivo model^35^, were grown at 34 °C until a 200–250 mg weight. The investigated complex was dispersed in PBS during the toxicity study using ultrasound (30 s, A = 18%, W = 250 W, on:off = 2:1 s) to form a 5 mg/ml stock solution. A 10 μl inoculum of the complex dispersion (in different concentrations) was injected into the upper right proleg area of the larvae using a Hamilton 22-gauge syringe. Each concentration of the complex was injected into five larvae per testing group (*n* = 5). The control group was inoculated with 10 μl of 1x PBS (Fisher Scientific) in the same manner. After inoculation, the larvae were kept at 37 °C for up to 72 h. Larval mortality was observed every 16–24 h. The testing was done repeatedly. The survival curves were plotted using Kaplan–Meier analysis, and statistically significant differences were determined by the one-sided log-rank test (GraphPad 9.0 Software).

### In vivo antibacterial efficacy

During the efficacy study, *G. mellonella* larvae were preinjected with 10 μl of an infective dose of *P. aeruginosa* (PAO1) (6.4 × 10^3^ cfu/ml) in the upper right proleg area. An hour after infection, a 10 μl inoculum containing different concentrations of the tested complex dispersed in PBS was injected into the upper left proleg. The following procedure was the same as for the toxicity study. The controls were larvae injected with 1x PBS (negative control) and larvae injected with ciprofloxacin (without complex) at 10 mg/kg (positive control). Each group tested contained five larvae (*n* = 5).

### Statistics and reproducibility

The experiments were done at least in triplicate and repeated 2–3 times (depend on the experiment). Results are presented as mean value ± SD. Differences between groups were assessed by the one-sided log-rank test (GraphPad 9.0 Software).

### Reporting summary

Further information on research design is available in the Nature Research Reporting Summary linked to this article.

## Supplementary information

Supplementary information

Description of Additional Supplementary Data

Supplementary Data 1

Supplementary Data 2

Supplementary Data 3

Supplementary Data 4

Supplementary Data 5

Supplementary Data 6

Supplementary Data 7

Supplementary Data 8

Supplementary Data 9

Supplementary Data 10

Reporting Summary-New

## Data Availability

All data generated or analyzed during this study are included in this published article and its supplementary information files. The source files behind Figs. 2, 4, 5, 6 and 9 are presented in Supplementary Data 1–5 files, respectively. The source files behind Supplementary Figs. 1–4 and 6 are provided in Supplementary Data 6–10 files, respectively.
