# Supplementary material for: Development of a ternary cyclodextrin–arginine–ciprofloxacin antimicrobial complex with enhanced stability
Source: Commun Biol. 2022 Nov 12;5:1234. doi: 10.1038/s42003-022-04197-9 (PMC9653501; doi:10.1038/s42003-022-04197-9)
Supplement: Supplementary file 1 — Supplementary information [file 42003_2022_4197_MOESM1_ESM.pdf]

Supplementary Information

Development of a ternary Cyclodextrin–Arginine–Ciprofloxacin antimicrobial complex with enhanced stability

M. Vukomanovic[1,2,*], L. Gazvoda[1,2], M. Kurtjak[1], J. Hrescak,[3] B. Jaklic,[1,2] L. Moya-Andérico[4], M. Cendra[4], E. Torrents[4,5,*]

[1]Advanced Materials Department, Institute Jozef Stefan, Jamova 39, Ljubljana, Slovenia

[2]International Postgraduate School of Jozef Stefan, Jamova 39, Ljubljana, Slovenia

[3]Center for Electronic Microscopy and Microanalysis (CEMM), Institute Jozef Stefan, Jamova 39, Ljubljana, Slovenia

[4]Bacterial Infections: Antimicrobial Therapies, Institute for Bioengineering of Catalonia (IBEC), The Institute of Science and Technology, Baldiri Reixac 15-21, 08028 Barcelona, Spain

[5]Microbiology Section, Department of Genetics, Microbiology and Statistics, Faculty of Biology, University of Barcelona, 643 Diagonal Ave., 08028, Barcelona, Spain

*Corresponding authors:

Dr. Eduard Torrents: etorrents@ibecbarcelona.eu

Dr. Marija Vukomanovic: marija.vukomanovic@ijs.si

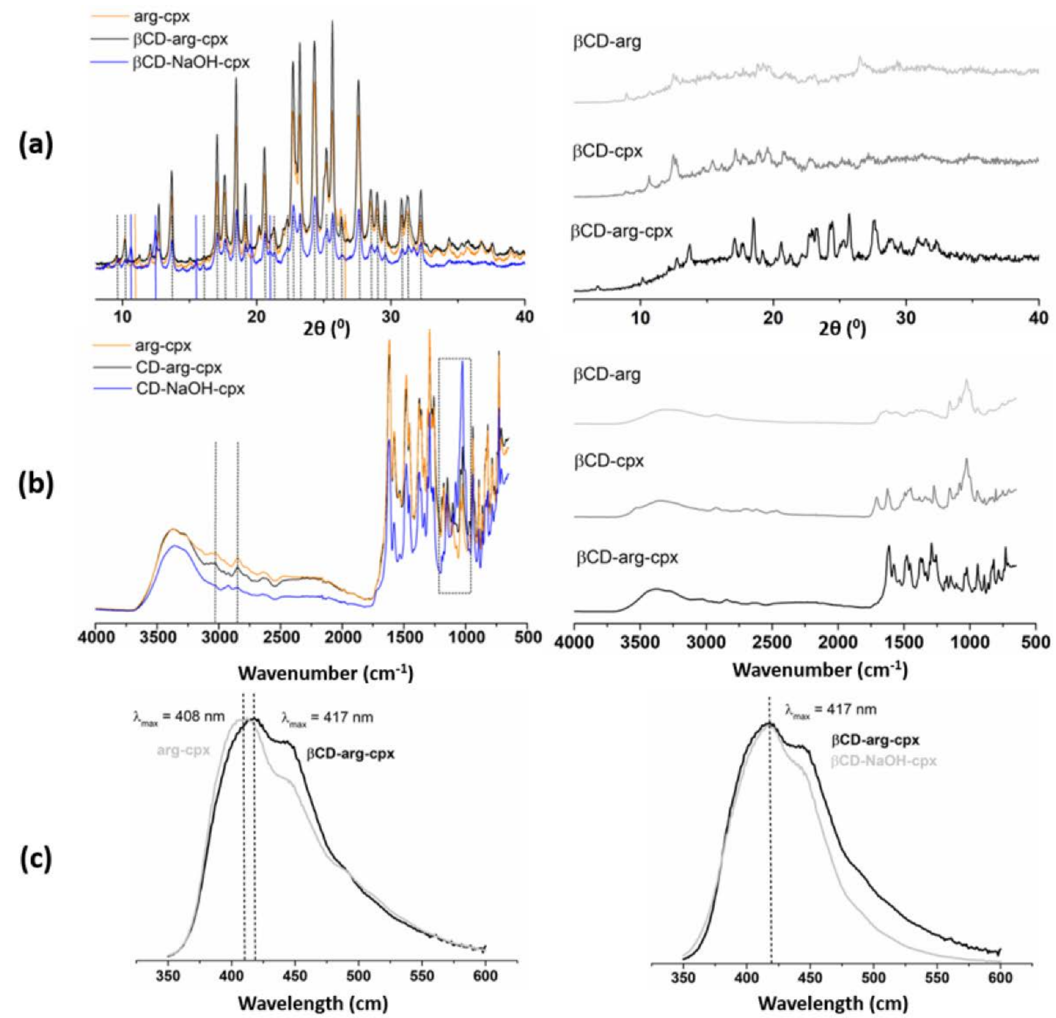

Supplementary Figure 1. Structural and chemical properties of CD-based complexes. Very similar crystal structures of arg-cpx, βCD-arg-cpx, and βCD-NaOH-cpx detected by XRD and their differences compared to βCD-arg and βCD-cpx (a). FTIR spectra showing differences in functional group vibrations in arg-cpx, βCD-arg-cpx, and βCD-NaOH-cpx, as well as their components (βCD-arg and βCD-cpx) (b). Position shifts detected in fluorescence spectra of cpx in complexes with and without βCD as well as the absence of the change in the case of cpx deprotonation with arg or NaOH (c).

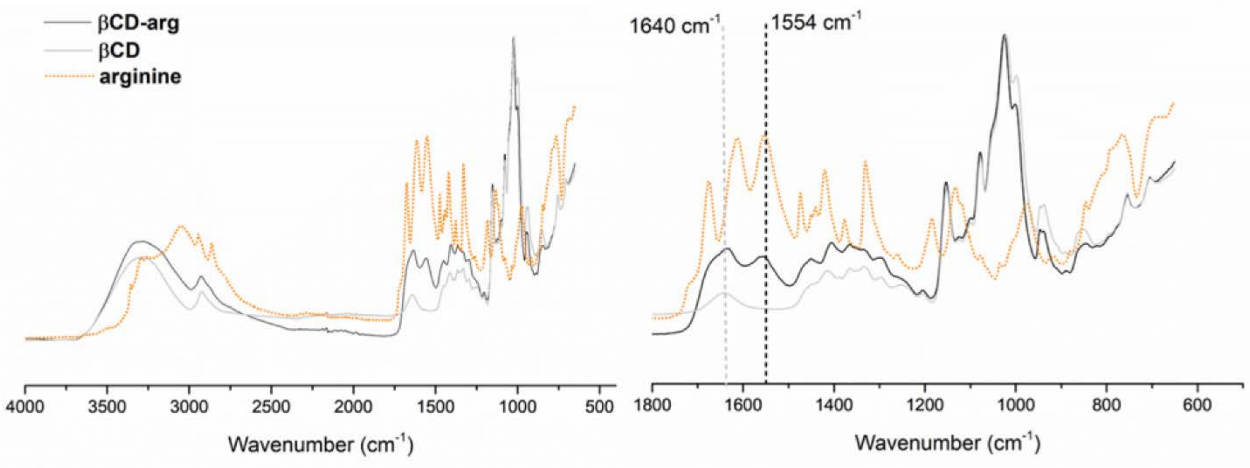

Supplementary Figure 2. FTIR spectra of the βCD-arg complex and its components arginine (arg) and cyclodextrin (βCD); annotated bands from βCD and arg components with increasing intensity within the complex.

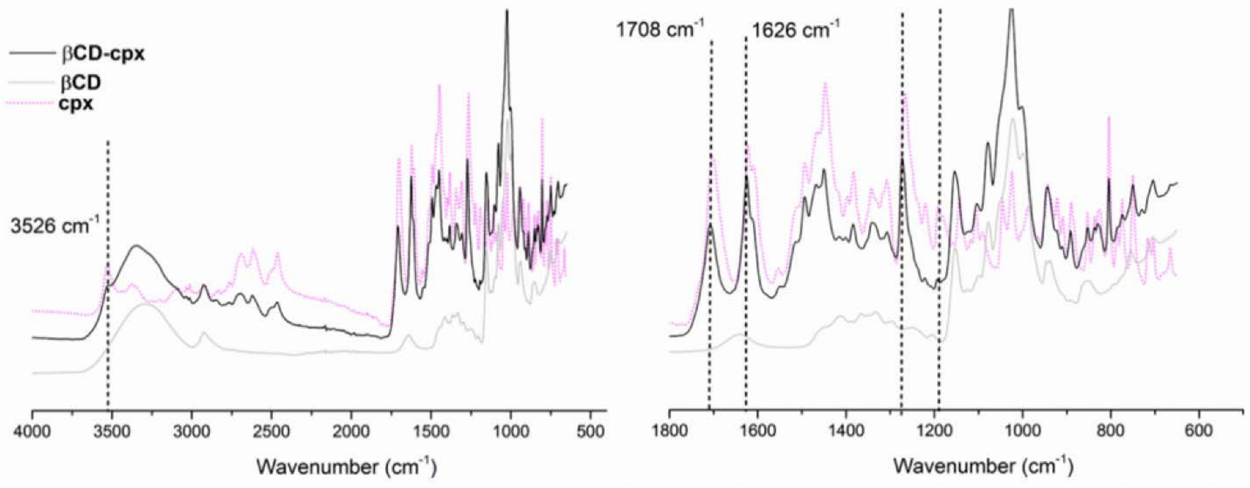

Supplementary Figure 3. FTIR spectra of the CD-cpx inclusion complex and its components ciprofloxacin (cpx) and cyclodextrin (CD); annotated bands of OH vibrations that also remain within the complex (absence of intermolecular hydrogen bonding) and shifts of the bands after complex formation due to interactions after incorporating cpx inside the CD cone.

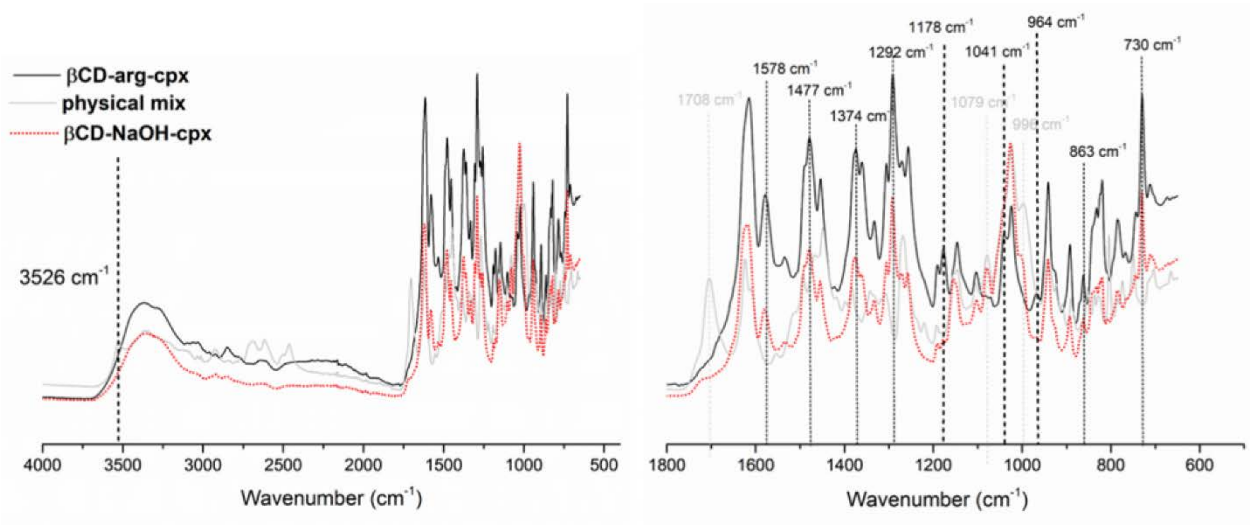

Supplementary Figure 4. FTIR spectra of βCD-arg-cpx more stable complex (with arginine linker), βCD-NaOH-cpx complex (with arginine replaced by NaOH), and physical mixture of complex components; bands of OH vibrations that disappear in complexed structures (due to deprotonation of a carboxyl group), novel vibrations (marked in black vertical dashed lines), and suppressed vibrations (marked in gray vertical lines) observed after βCD-arg-cpx and βCD-NaOH-cpx complex formation are annotated. Bold black dashed lines annotate bands characteristic of βCD-arg-cpx that do not exist in the βCD-NaOH-cpx spectrum, identifying differences in their structures.

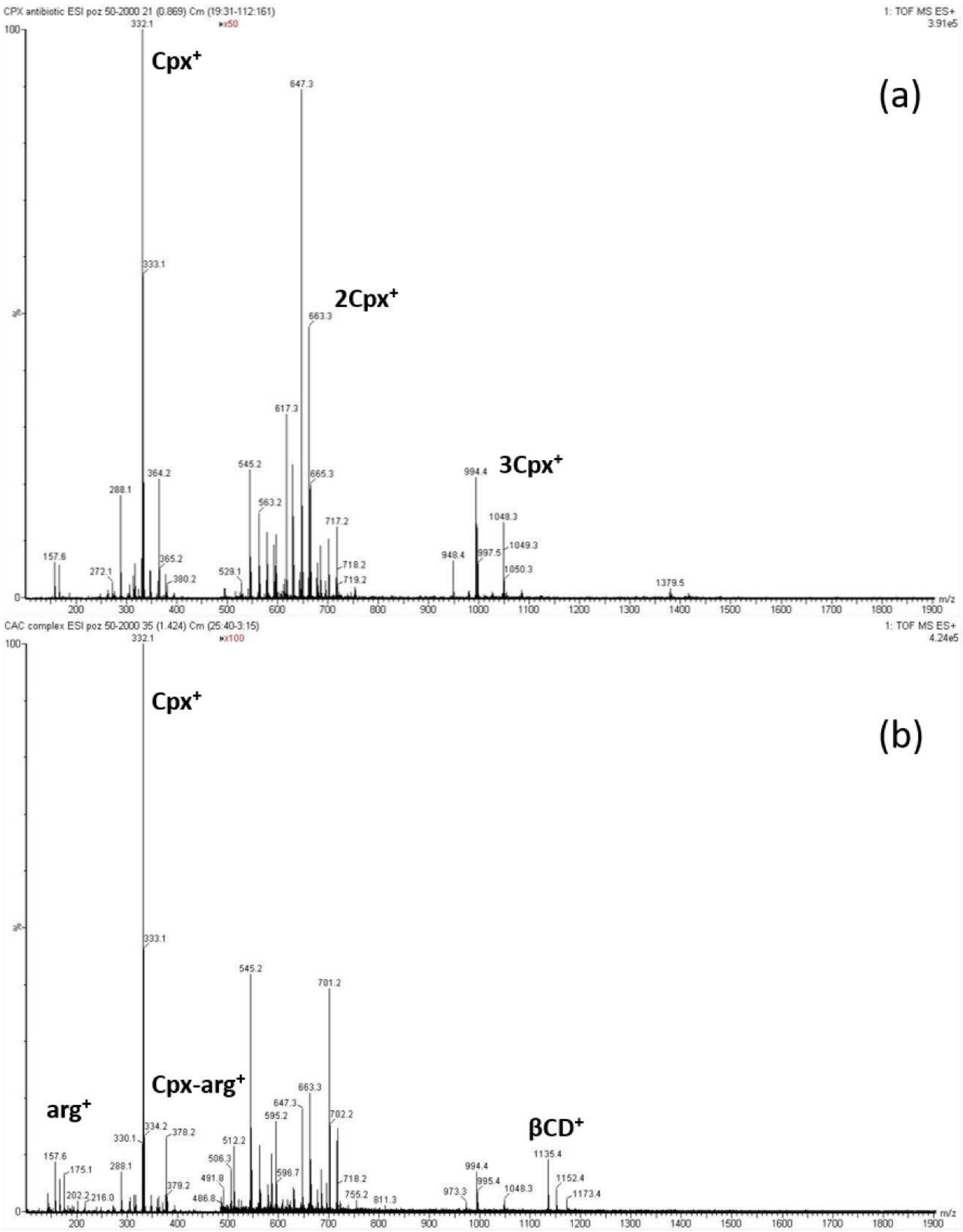

Supplementary Figure 5. Mass spectra of components released in water after complete dissolving cpx (a) and partial dissolving of βCD-arg-cpx complex (b) during 24-h aging in aqueous environment.

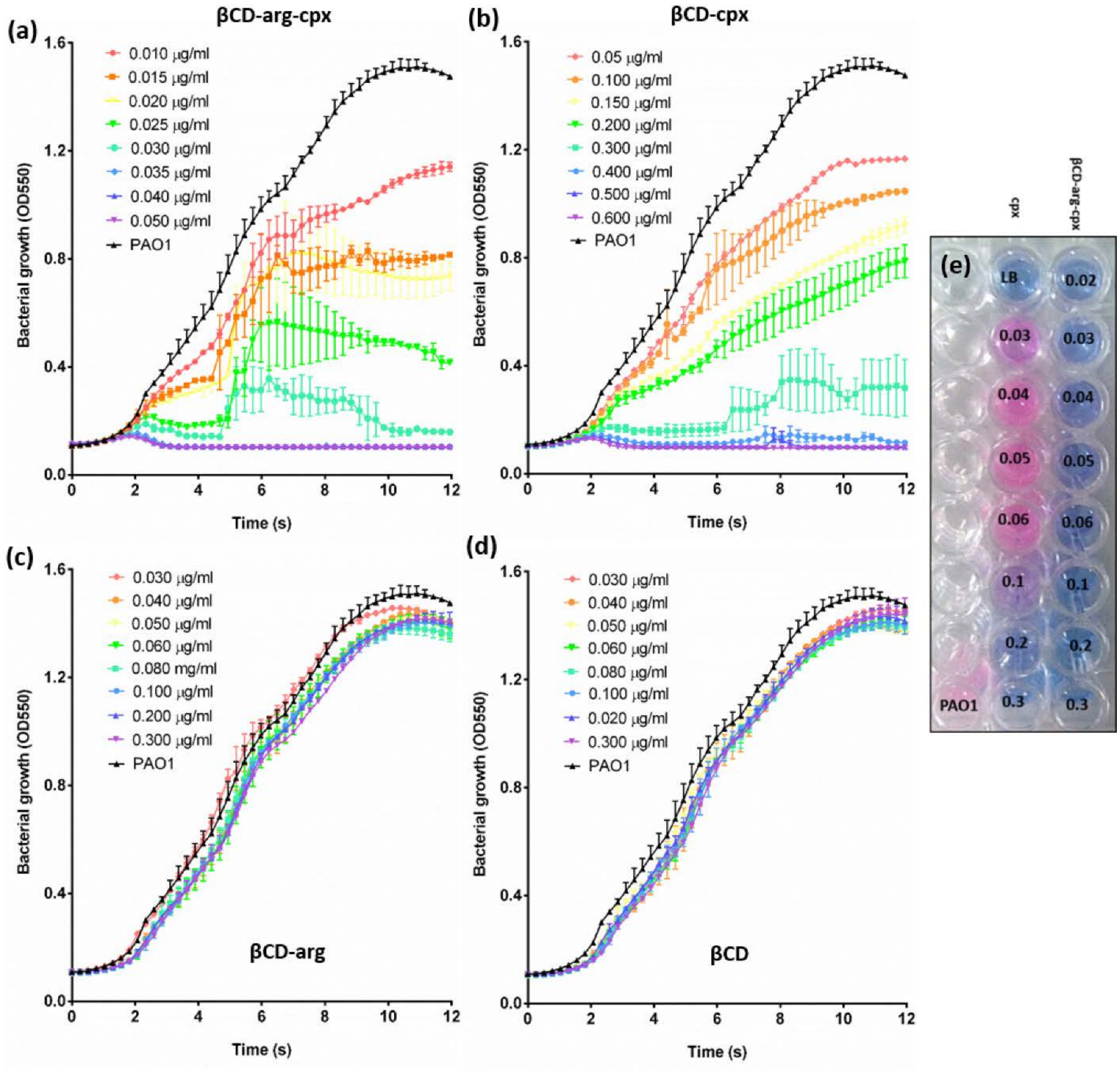

Supplementary Figure 6. Microdilution test showing kinetics of PAO1 growth in presence of βCD-arg-cpx (0.010-0.050 μg/ml) (a), βCD-cpx (0.050-0.60 μg/ml) (b), βCD-arg (0.030-0.300 μg/ml) (c) and βCD (0.030-0.300 μg/ml) (d) as well as Presto blue test showing direct comparison between activity of βCD-arg-cpx and free cpx drug (e); n=3, error bars refer to CD of optical density.

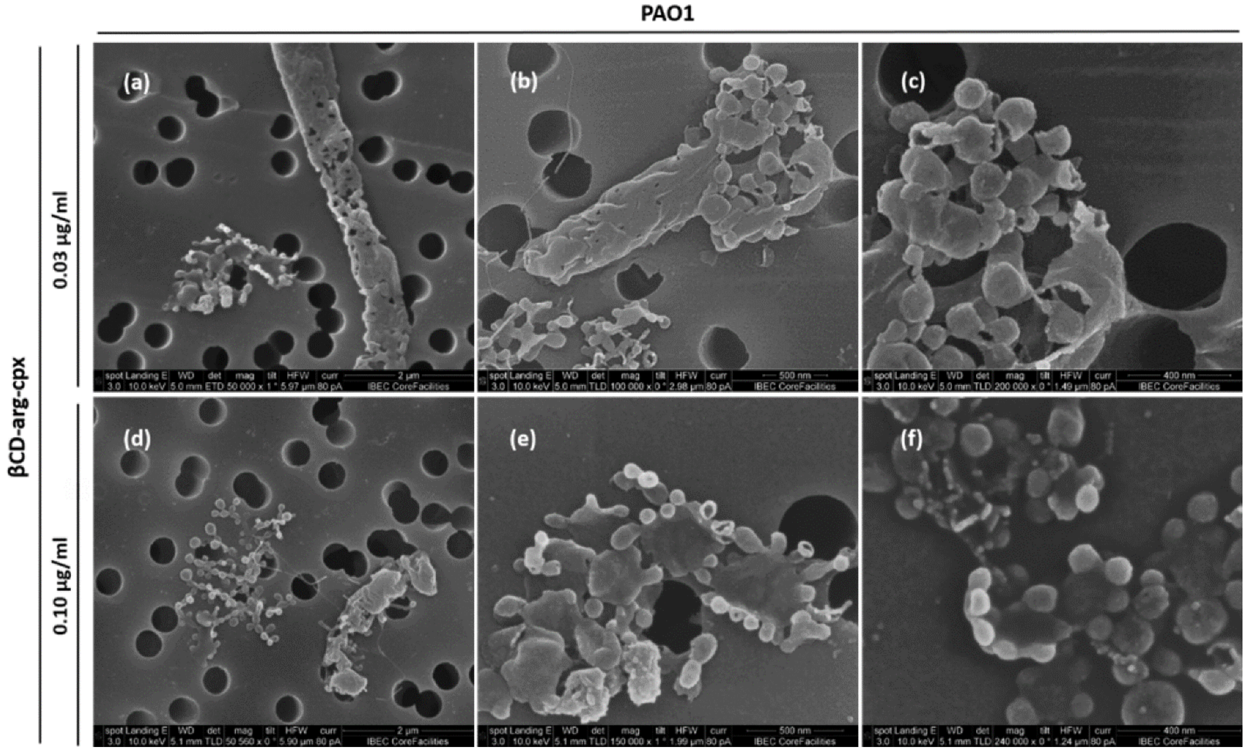

Supplementary Figure 7. Decomposition of membrane into vesicle-like structures observed in *P. aeruginosa* PAO1 for filamented and normal-sized cells (βCD-arg-cpx concentration 0.03 µg/ml (0.02 µM)) (a-c) and only nonfilamented bacteria (βCD-arg-cpx concentration 0.1 µg/ml (0.06 µM)) (d-f).

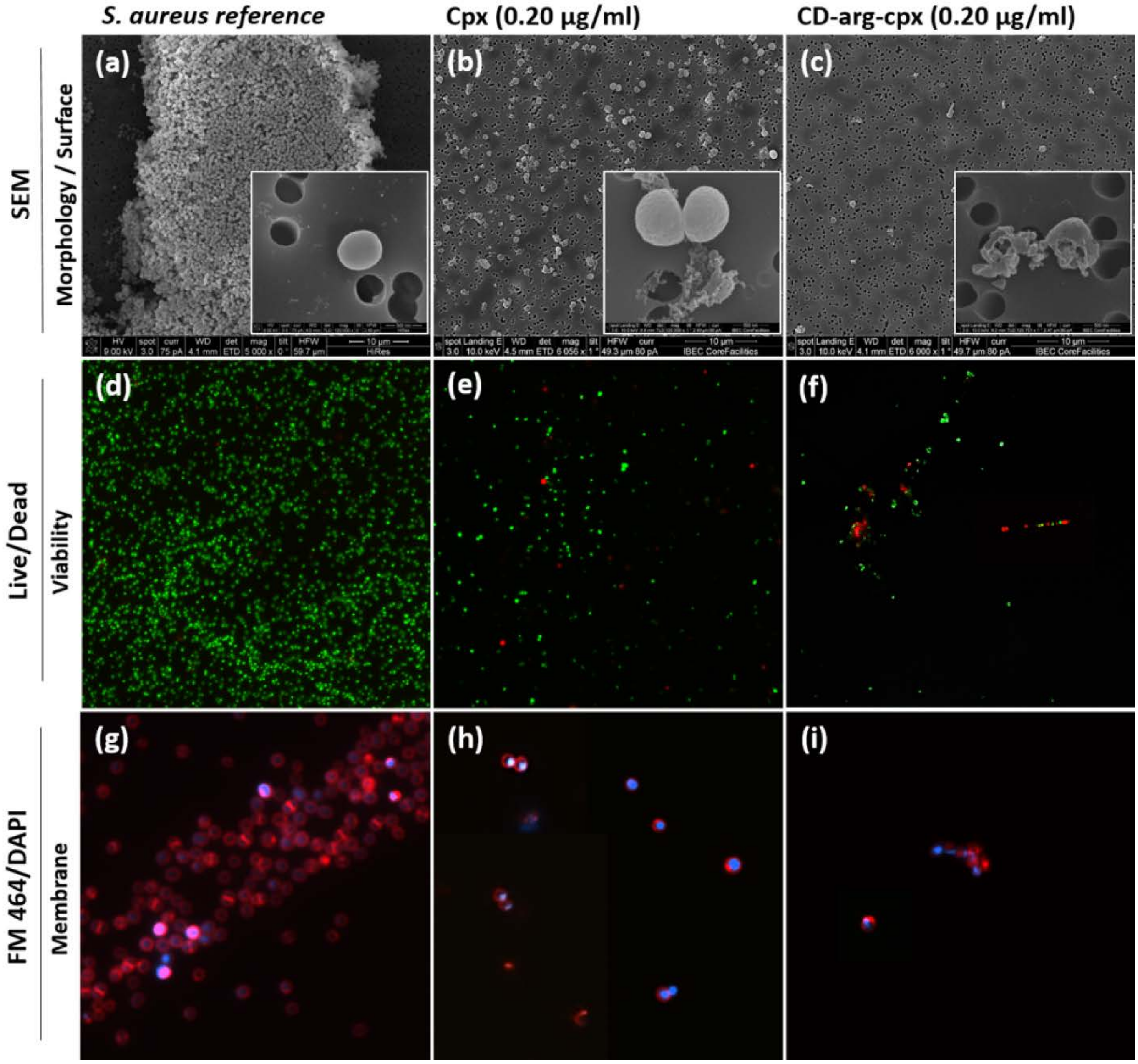

Supplementary Figure 8. *S. aureus* exposed to ciprofloxacin and its βCD-arg complex. Morphology and surface of *S. aureus* before (a) and after exposure to low cpx concentration (0.20 µg/ml) (b) and equivalent cpx in βCD-arg-cpx (c) cells; live *S. aureus* stained with Live/Dead dyes (d) and fraction of viable cells treated with 0.03 µg/ml cpx (e) and βCD-arg-cpx (f); membrane structure of wild-type *S. aureus* stained with FM464/DAPI dyes (g) and in bacteria treated with 0.03 µg/ml of cpx (h) and βCD-arg-cpx complex (i).
