# Supplementary material for: Development of a ternary cyclodextrin–arginine–ciprofloxacin antimicrobial complex with enhanced stability
Source: Commun Biol. 2022 Nov 12;5:1234. doi: 10.1038/s42003-022-04197-9 (PMC9653501; doi:10.1038/s42003-022-04197-9)
Supplement: Supplementary file 2 — Description of Additional Supplementary Data [file 42003_2022_4197_MOESM2_ESM.docx]

**Description of Additional Supplementary Files**

**File name:** Supplementary Data 1

**Description:** The source data behind the XPS spectra in Figure 2.

**File name:** Supplementary Data 2

**Description:** The source data behind the fluorescence spectra in Figure 4.

**File name:** Supplementary Data 3

**Description:** The source data behind the UV/vis spectra and drug release study in

**File name:** Supplementary Data 4

**Description:** The source data behind the in vitro toxicity and comparative antimicrobial activity in Figure 6.

**File name:** Supplementary Data 5

**Description:** The source data behind in vivo toxicity and antimicrobial efficacy in Figure 9.

**File name:** Supplementary Data 6

**Description:** The source data behind diffractograms, FTIR and fluorescence spectra

**File name:** Supplementary Data 7

**Description:** The source data behind FTIR spectra in Supplementary Figure 2.

**File name:** Supplementary Data 8

**Description:** The source data behind FTIR spectra in Supplementary Figure 3.

**File name:** Supplementary Data 9

**Description:** The source data behind FTIR spectra in Supplementary Figure 4.

**File name:** Supplementary Data 10

**Description:** The source data behind kinetics of antimicrobial action against P. aeruginosa in Supplementary Figure 6.
